# Hedgehog interacting protein–expressing lung fibroblasts suppress lymphocytic inflammation in mice

**DOI:** 10.1172/jci.insight.144575

**Published:** 2021-09-08

**Authors:** Jeong H. Yun, ChangHee Lee, Tao Liu, Siqi Liu, Edy Y. Kim, Shuang Xu, Jeffrey L. Curtis, Luca Pinello, Russell P. Bowler, Edwin K. Silverman, Craig P. Hersh, Xiaobo Zhou

**Affiliations:** 1 Channing Division of Network Medicine, and; 2 Division of Pulmonary and Critical Care, Department of Medicine, Brigham and Women’s Hospital, Boston, Massachusetts, USA.; 3 Harvard Medical School, Boston, Massachusetts, USA.; 4 Department of Genetics, Blavatnik Institute, Harvard Medical School, Boston, Massachusetts, USA.; 5 Department of Internal Medicine, University of Michigan Medical School & Michigan Medicine, Ann Arbor, Michigan, USA.; 6 VA Ann Arbor Healthcare System, Ann Arbor, Michigan, USA.; 7 Department of Pathology, Massachusetts General Hospital, Boston, Massachusetts, USA.; 8 Department of Medicine, National Jewish Health, Denver, Colorado, USA.

**Keywords:** Inflammation, Pulmonology, COPD, Mouse models

## Abstract

Chronic obstructive pulmonary disease (COPD) is mainly caused by cigarette smoking and characterized by chronic inflammation in vulnerable individuals. However, it is unknown how genetic factors may shape chronic inflammation in COPD. To understand how hedgehog interacting protein, encoded by *HHIP* gene identified in the genome-wide association study in COPD, plays a role in inflammation, we utilized *Hhip^+/–^* mice that present persistent inflammation and emphysema upon aging similar to that observed in human COPD. By performing single-cell RNA sequencing of the whole lung from mice at different ages, we found that *Hhip^+/–^* mice developed a cytotoxic immune response with a specific increase in killer cell lectin-like receptor G1–positive CD8^+^ T cells with upregulated *Ifn****γ* expression recapitulating human COPD. *Hhip* expression was restricted to a lung fibroblast subpopulation that had increased interaction with CD8^+^ T lymphocytes in *Hhip^+/–^* compared with *Hhip^+/+^* during aging. Hhip-expressing lung fibroblasts had upregulated IL-18 pathway genes in *Hhip^+/–^* lung fibroblasts, which was sufficient to drive increased levels of IFN-**γ** in CD8^+^ T cells ex vivo. Our finding provides insight into how a common genetic variation contributes to the amplified lymphocytic inflammation in COPD.

## Graphical abstract

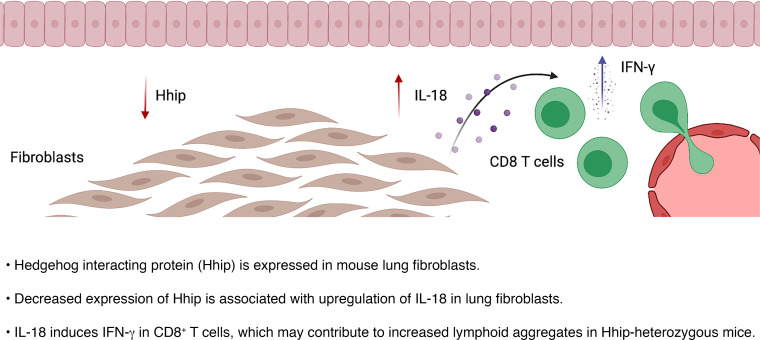

## Introduction

Chronic obstructive pulmonary disease (COPD) is a highly prevalent chronic inflammatory lung disease characterized by progressive airflow limitation. Cigarette smoking is the major risk factor for COPD. However, there is marked heterogeneity in disease susceptibility and clinical manifestations of COPD among smokers with similar smoking histories. Genetic factors have been identified to account for these variations, but the cellular and molecular basis of genetic susceptibility has not been fully understood. Variable inflammation among smokers, including infiltration and activation of immune cells, possibly attributable to genetic risks of COPD, are amplified in patients with COPD compared with asymptomatic smokers.

The hedgehog interacting protein (*HHIP*) locus on chromosome 4q31 is one of the most replicated loci, strongly associated with COPD in multiple genome-wide association studies (GWAS) (1–4). It has been associated with severity and distribution of emphysema (5, 6) as well as with lung function, measured as forced expiratory volume in 1 second (FEV_1_) and FEV_1_/forced vital capacity (FVC), in both smokers and nonsmokers (1, 7, 8). We have previously found COPD risk alleles at the *HHIP* locus to be associated with reduced distal enhancer activity, suggesting lower levels of *HHIP* increase the risk for COPD (9). *HHIP* negatively regulates the hedgehog signaling pathway by competitively binding with hedgehog ligands. *Hhip*-knockout mice develop hypoplastic lungs due to defective branching morphogenesis, leading to postnatal lethality (10). However, *Hhip*-haploinsufficient (*Hhip^+/–^*) mice develop normal lungs, histologically indistinguishable from WT controls (11). We found that with an approximately 30% reduction of HHIP mRNA levels in lungs, *Hhip^+/–^* mice display not only more severe emphysema, but also prominent airway lymphoid aggregates after chronic cigarette smoke (CS) exposure, characteristic of severe COPD (12). Furthermore, *Hhip^+/–^* mice form evident lymphoid aggregates as they age, even in the absence of CS exposure, suggesting that *Hhip^+/–^* mice have inherent susceptibility toward exaggerated inflammation, a key feature of human COPD lungs that was not recapitulated in other murine models of COPD (13). Although HHIP was shown to be expressed in mesenchyme during murine lung development (10), it remains unclear which cell types express HHIP after lung development and how haploinsufficiency of HHIP results in lymphocytic inflammation.

To address these questions, we investigated associations between HHIP variant and COPD phenotypes in participants in the COPDGene Study and assessed the impact of *Hhip* haploinsufficiency in murine lungs at different age points by single-cell RNA sequencing. Our study suggests that *Hhip*, expressed in murine lung fibroblasts, represses CD8^+^ T cell activation, possibly through IL-18. Insufficient levels of Hhip result in expansion of dysregulated and activated CD8^+^ T cells, contributing to lymphocytic inflammation. In human lungs, HHIP is expressed in both mesenchymal and epithelial compartments. Patients with COPD carrying the HHIP risk allele display elevated IL-18 levels and increased COPD exacerbation frequency. These findings provide mechanistic insights into chronic inflammation seen in people who are genetically susceptible to COPD.

## Results

### HHIP risk variant is associated with COPD exacerbations.

HHIP variant rs1032296 (risk allele T) has been associated with lung function in smokers (14) and patients with COPD (1). In 6627 non-Hispanic White former and current smokers in the COPDGene Study, we confirmed the COPD risk variant to be associated with COPD (FEV_1_/FVC < 0.7) and lower FEV_1_. Furthermore, the risk variant was also associated with increased emphysema, peripheral blood WBC counts, and fibrinogen levels (Table 1). As frequent COPD exacerbation is associated with persistent airway and systemic inflammation (15), we examined whether *HHIP* COPD risk variant is also associated with COPD exacerbation frequency. In a subset of patients with COPD from Table 1 (*n* = 3405), HHIP risk allele rs1032296 (T) was associated with increased exacerbations in the year prior to enrollment (incidence rate ratio 1.08, *P* = 0.046) in multivariable analysis (adjusted for age, sex, baseline St. George’s Respiratory Questionnaire score, FEV_1_ percent predicted, gastroesophageal reflux disease, and genetic ancestry principal components) (Table 2).

### Single-cell RNA sequencing of murine lungs identifies expression of Hhip in fibroblasts.

**Figure 1:**
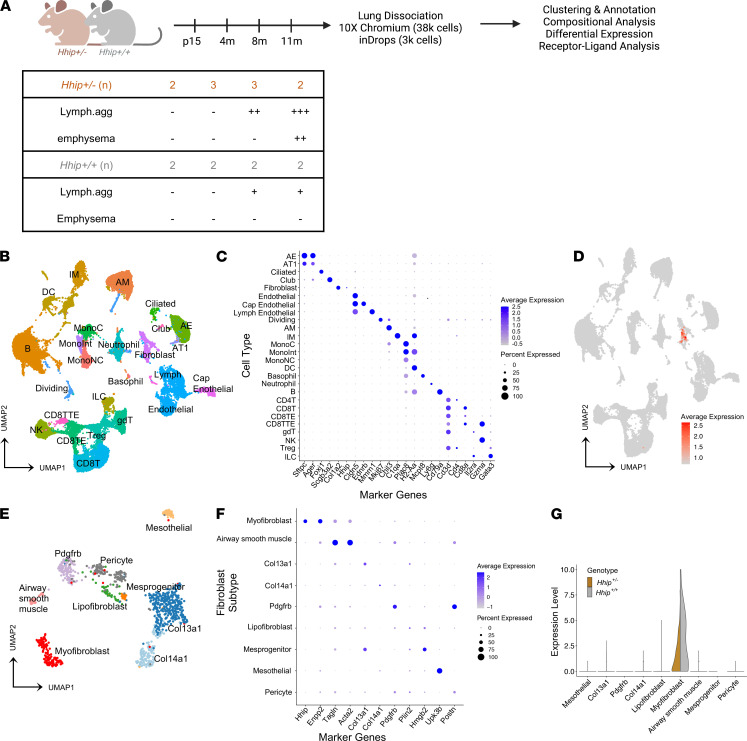
Single-cell RNA sequencing of Hhip^+/–^ and Hhip^+/+^ murine lungs. (**A**) Experimental design of lung single-cell RNA sequencing from age-matched *Hhip^+/–^* and WT littermate *Hhip^+/+^* mice. (**B**) Unbiased clustering of 38,875 cells from 26 clusters by uniform manifold approximation and projection (UMAP) plot. (**C**) Expression of representative marker genes across cell type clusters. (**D**) UMAP plot indicating fibroblast cluster expressing *Hhip* (red dots). (**E**) Nine subclusters of lung fibroblasts showing heterogeneity. (**F**) Expression of representative fibroblast subcluster marker genes shown in dot plot. *Hhip* is a marker gene for myofibroblast subcluster. (**G**) Hhip expression is decreased in *Hhip^+/–^* lungs. Expression values are normalized unique molecular identifier (UMI) count. Lymph.agg, lymphoid aggregates; AE: alveolar epithelial cell; AT1, type 1 alveolar epithelial cell; Cap Endothelial, capillary endothelial cell; Lymph Endothelial, lymphatic endothelial cell; AM, alveolar macrophage; IM, interstitial macrophage; Mesprogenitor: mesenchymal progenitor; MonoC, classical monocyte; MonoInt, intermediate monocyte; MonoNC, nonclassical monocyte; DC, dendritic cell; CD8TE, CD8^+^ effector memory T cell; CD8TTE, CD8^+^ terminal effector T cell; gdT, γδ T cell; NK, natural killer cell; Treg, regulatory T cell; ILC, innate lymphoid cell.****

Resembling the persistent inflammation in human COPD lungs, prominent lymphoid aggregates are exhibited in *Hhip^+/–^* mice upon CS exposure and aging (11, 13). However, quantitative PCR (qPCR) analysis of sorted lung cell types indicated that *Hhip* was highly expressed in fibroblasts instead of immune or epithelial cells (Supplemental Figure 1; supplemental material available online with this article; https://doi.org/10.1172/jci.insight.144575DS1). To confirm this and determine molecular impacts of Hhip haploinsufficiency in lungs, we performed single-cell RNA sequencing, primarily using the 10x Chromium (v2) platform in lungs from *Hhip*^+/–^ mice and WT littermates at 4 age points (13) (Figure 1A). These time points represent late development (postnatal 15 days, P15), an early adult stage with normal histology (4 months), middle age with the development of lymphoid aggregates (8 months), and advanced age with emphysema and substantial lymphoid aggregates (11 months). A total of 38,875 cells passing quality control were analyzed (Supplemental Table 1). To account for experimental and technical variability, we used Seurat (version 3.1.4) integrative analysis with SCTransform to identify shared cell states and populations across different data sets and platforms (16) (Supplemental Figure 2). Using graph-based clustering (17), 26 clusters of cells were identified, encompassing all epithelial, mesenchymal, immune (lymphoid and myeloid), and endothelial cell groups (Figure 1, B and C; Supplemental Figure 3A; and Supplemental Data File 1). The graph-based clusters were manually annotated with differentially expressed genes and validated by projection of previously published single-cell RNA-sequencing atlases (18, 19). Additionally, we annotated T cell subtypes by utilizing the ImmGen database (20), and manually annotated natural killer T (NKT) cells, which were mostly grouped within regulatory T cells (Supplemental Figure 3, B and C).

*Hhip* expression was restricted to the mesenchymal population characterized by expression of collagen (*Col3a1*, *Col1a2*) and smooth muscle actin (*Acta2*) and was absent from other clusters, including the immune cell clusters (Figure 1D). These mesenchymal cells were further categorized by subcluster analysis into 9 subclusters by reference-based annotation (19), including matrix fibroblasts, myofibroblasts, lipofibroblasts, and mesothelial population (Figure 1E). *Hhip* was a top marker gene for the myofibroblast subcluster (AUC 0.81, adjusted *P* < 3 × 10^–104^), which also expressed gremlin 2, DAN family BMP antagonist (*GREM2*); ectonucleotide pyrophosphatase/phosphodiesterase (*ENPP1*); and *Acta2* (19) (Figure 1F and Supplemental Data File 1). As expected, *Hhip* expression was reduced in *Hhip^+/–^* lung fibroblasts (Figure 1G).

### Number of CD8^+^ terminal effector cells is increased with aging in lungs from Hhip^+/–^ mice.

**Figure 2:**
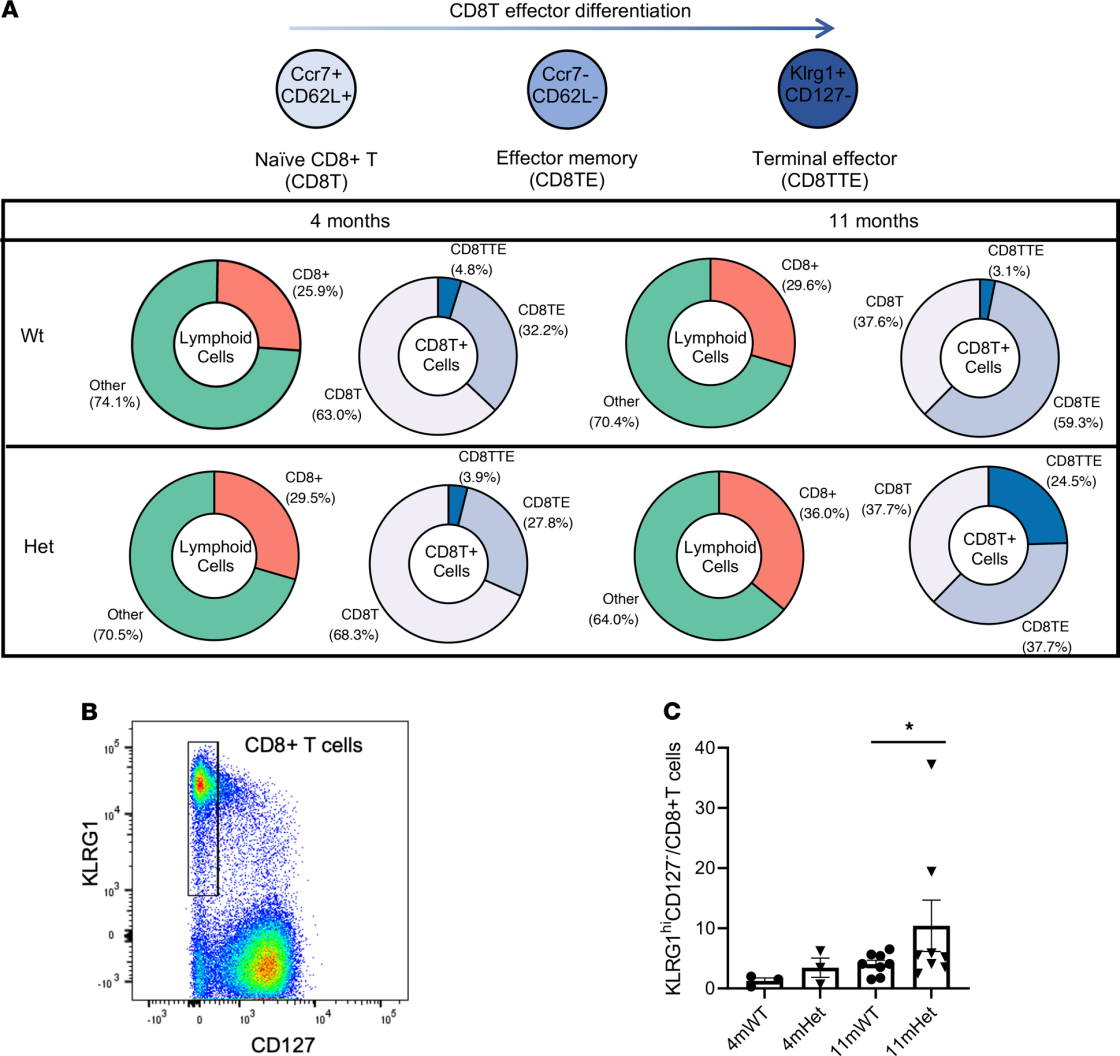
Number of CDTTEs increased in Hhip^+/–^ mice with age. (**A**) Naive CD8^+^ T cells (Ccr7^+^CD62L^+^) differentiate into terminal effector cells (Klrg1^+^CD127^–^). Proportion of CD8^+^ T cells including naive CD8^+^ T (CD8T) cells, effector memory CD8^+^ T cells (CD8TE), and terminal effector CD8^+^ T cells (CD8TTE) increases with age in *Hhip^+/–^* (Het) mice. (**B**) FACS gating strategy for CD8TTE cells. CD8^+^ T cells were gated for KLRG1^hi^CD127^–^ populations. (**C**) Proportion of CD8TTEs in total CD8^+^ T cells. Each point represents an individual biological replicate. Error bars indicate standard error of the mean (SEM). *P < 0.05. Wilcoxon matched pairs (by age- and sex-matched littermates) signed rank test.

Notably, the proportion of CD8^+^ T cells among lymphoid cells in *Hhip^+/–^* mice increased with age (Figure 2), while the relative fraction of CD4^+^ T cells among lymphoid cells decreased (Supplemental Figure 4), concomitant with the appearance of lung lymphoid aggregates in *Hhip^+/–^* mice. Moreover, among CD8^+^ T cell subtypes, we found an increase of the percentage of specific CD8^+^ T cell subgroup marked by expression of killer cell lectin-like receptor G1 (*Klrg1*) in *Hhip^+/–^* mice at 11 months of age from 3.1% to 24.5% while percentage of other lymphoid cell subtypes showed minimal genotypic differences (Figure 2A and Supplemental Figure 4). Klrg1 is induced in highly cytotoxic and proliferative CD8^+^ T effector cells and serves as a marker for terminal differentiation of CD8^+^ T cells (21). The expanded CD8^+^ T cell population expressed additional effector T cell markers Cx3cr1, Tbx21, and Gzma but lacked expression of memory T cell markers, such as CD127 (Il7r), Cd27, and Tcf7, corresponding to CD8TTEs, or short-lived effector T cells (22) (Supplemental Figure 5A). FACS analysis in lungs from mice at 11 months of age confirmed increased proportion of CD8TTE cells marked by KLRG1^hi^CD127^–^ as well as increased levels of KLRG1 in CD8TTEs in *Hhip^+/–^* mice (Figure 2, B and C; and Supplemental Figure 5, B and C).

### CD8TTEs have increased expression of Ifnγ in Hhip^+/–^ mice.

**Figure 3:**
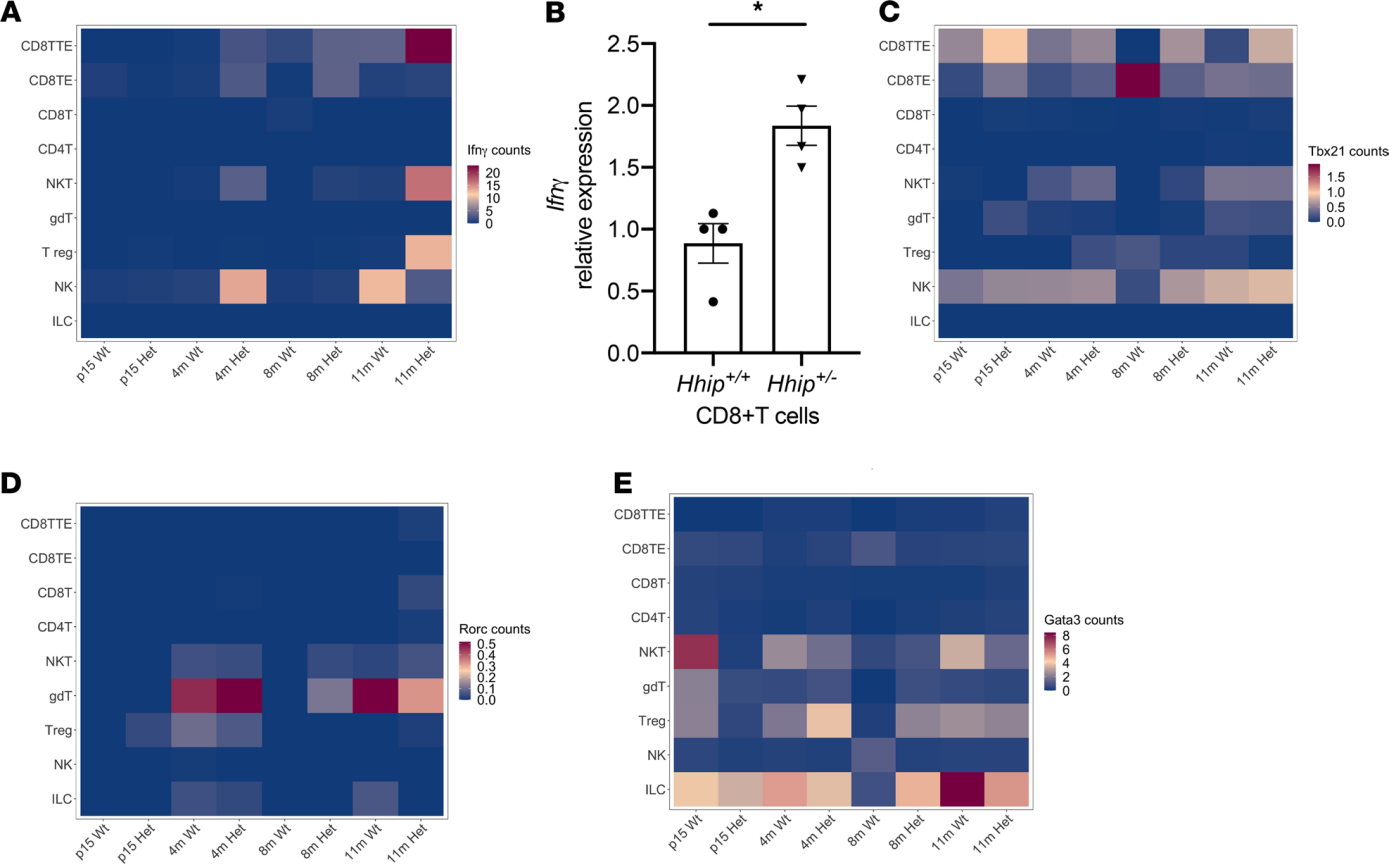
Increased activation of CDTTEs in Hhip+/– mice. (**A**) Average expression (normalized UMI count) of *Ifnγ* in various lymphocyte subtypes showing terminal effector CD8^+^ T (CD8TTE) cells with increased *Ifnγ* expression in *Hhip^+/–^* mice. (**B**) *Ifnγ* expression is increased in *Hhip^+/–^* lung CD8^+^ T cells as measured by qPCR (*n* = 4 mice/group, 4 months of age). Error bars indicate SEM. (**C**) Average expression of *Tbx21* in various lymphocytes showing CD8TTE cells with increased *Tbx21* expression in *Hhip^+/–^* mice. (**D**) Average expression of *Rorc* in various lymphocyte subtypes. (**E**) Average expression of *Gata3* in various lymphocytes with enrichment in Tregs, NKT cells, and ILCs. *P < 0.05, unpaired Student’s *t* test. CD8TTE, CD8^+^ terminal effector T cell; CD8TE, CD8^+^ effector memory T cell; CD8T, CD8^+^ naive T cell; CD4T, CD4^+^ T cell; NKT, natural killer T cell; gdT, γδ T cell; Treg, regulatory T cell; NK, natural killer cell; ILC, innate lymphoid cell.

Cell type–specific differential gene expression analysis showed increased expression of *Ifnγ* in combined CD8^+^ T cells from *Hhip^+/–^* mice (Wilcoxon rank sum test, adjusted *P* < 0.05, Figure 3A). We confirmed increased expression of *Ifnγ* in CD8^+^ T cells from the lungs of 4-month-old *Hhip^+/–^* mice by qPCR (Figure 3B). This is most likely driven by CD8TTE, the only immune cell type showing age-dependent increases in *Ifnγ* UMI counts in *Hhip^+/–^*, in contrast to other cell types that can also produce IFN-γ (Figure 3A). Furthermore, expression of *Tbx21*, a key transcription factor inducing expression of *Ifnγ*, was increased in *Hhip^+/–^* CD8TTEs compared with age-matched *Hhip^+/+^* cells as early as 15 days of age (Figure 3C), suggesting the activation of the Tbx21-*Ifnγ* in *Hhip^+/–^* CD8TTE cells precedes emphysema and lymphocytic aggregates’ formation in *Hhip^+/–^* mice. In contrast, expression of transcription factors important for Th17 (*Rorc*) and Th2 (*Gata3*) cell differentiation showed no consistent genotypic difference in a given T cell subtype (Figure 3, D and E).

### Pathway analysis on cell type specific gene expression changes suggest fibroblast-immune cell interactions.

**Figure 4:**
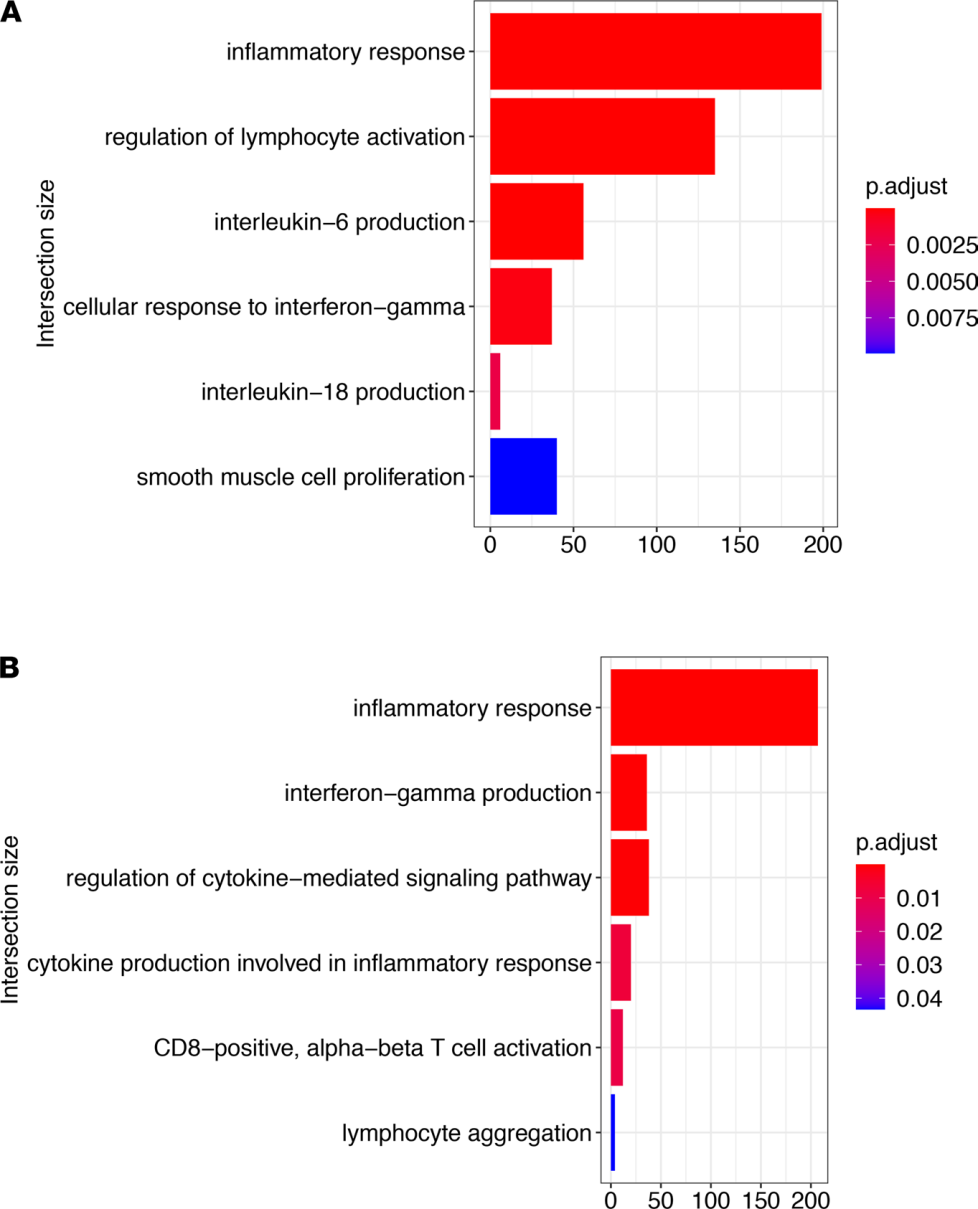
Inflammatory response–related pathways are enriched in Hhip^+/–^ CD8+ T cells and fibroblasts. Selected pathways that are significantly enriched with upregulated genes in *Hhip^+/–^* compared with *Hhip^+/+^* mice by Gene Ontology (GO) analysis in (**A**) all CD8^+^ T cells and (**B**) fibroblasts. Pathway analysis was performed with gprofiler2. *P* value is FDR adjusted.

Cell type-specific pathway analysis on upregulated genes in *Hhip^+/–^* lungs indicates significant enrichment of inflammatory and immune pathways in fibroblasts and immune cells at as early as 4 months of age, which may drive subsequently increased lymphocytic inflammation with age-associated emphysema in *Hhip^+/–^* mice at 10 months of age (13) (Supplemental Figure 6 and Supplemental Data File 2). In *Hhip^+/–^* CD8^+^ T cells, the IFN-γ production pathway was enriched (Figure 4A), supporting increased expression of IFN-γ (Figure 3, A and B) in CD8^+^ T cells. Interestingly, upregulated genes in *Hhip^+/–^* fibroblasts were enriched for pathways related to interaction with lymphocytes, including lymphocyte activation and IL-18 production, an IFN-γ–inducing cytokine (Figure 4B).

**Figure 5:**
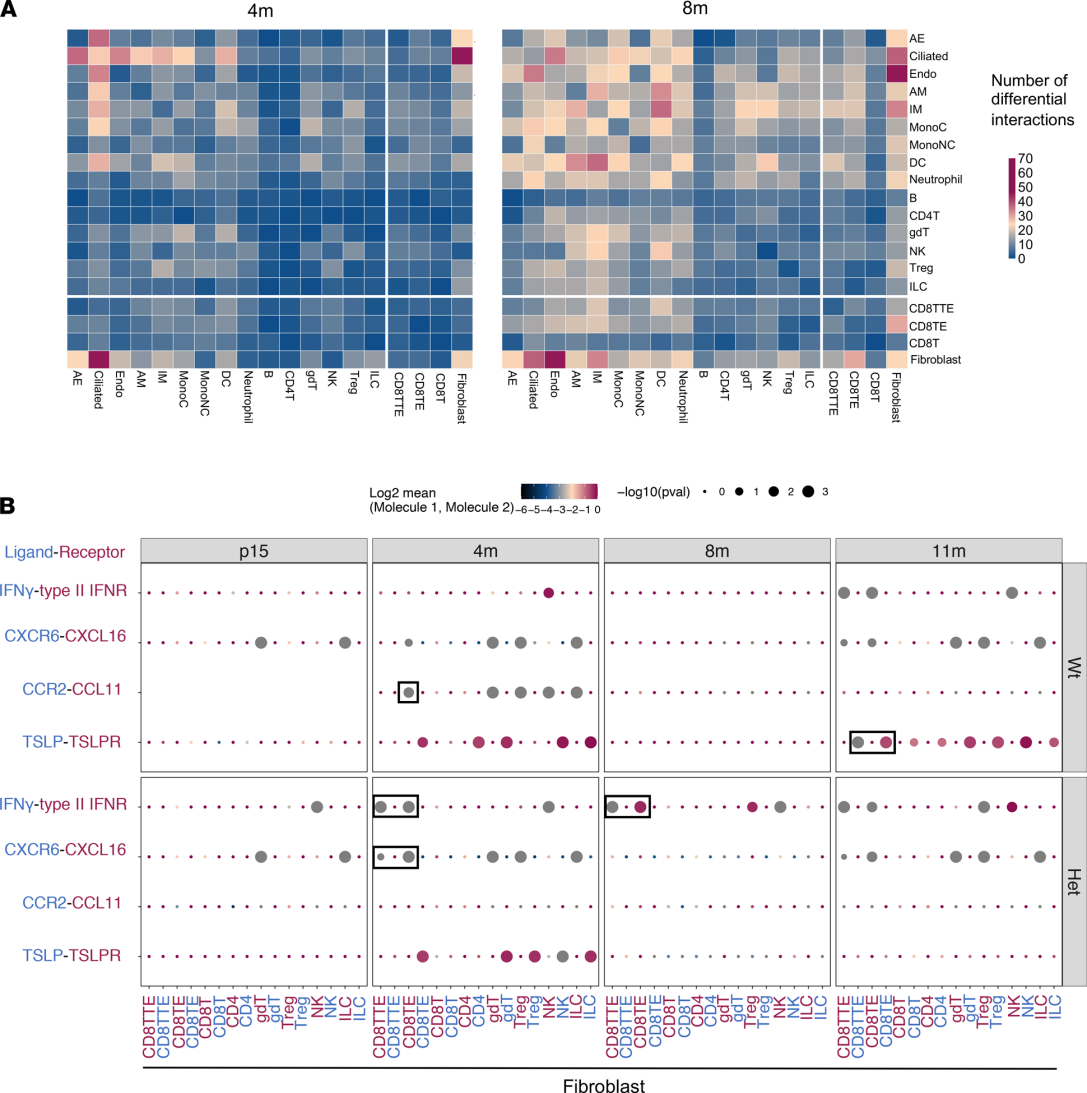
Receptor-ligand analysis suggests dysregulated fibroblast-T cell interaction in lungs from *Hhip*-haploinsufficient mice. (**A**) Genotype-dependent differential receptor-ligand interactions across various cell types from mice at 4 months (4m) and 8 months (8m) of age. Color scale bar indicates number of interaction pairs between cell types. (**B**) Selected ligand (blue)-receptor (pink) pairs between fibroblasts and immune cells from mice at different age points. Select receptor-ligand interaction pairs that are different by genotype are marked by black rectangles. Dot size indicates *P* values and the means of expression levels (normalized UMI counts) of interacting molecules are indicated by color of each dot. Wt, *Hhip^+/+^*; Het, *Hhip^+/–^*; AE, alveolar epithelial cell; Endo, endothelial cell; AM, alveolar macrophage; IM, interstitial macrophage; MonoC, classical monocyte; MonoNC, nonclasscial monocyte; DC, dendritic cell; B, B cell; CD8T, CD8^+^ naive T cell; CD8TE, CD8^+^ effector memory T cell; CD8TTE, CD8^+^ terminal effector T cell; gdT, γδ T cell; NK, natural killer cell; Treg, regulatory T cell; ILC, innate lymphoid cell.

Cell-cell communication, such as fibroblast-CD8^+^ T cell crosstalk, commonly occurs through receptor-ligand interaction. Therefore, we further investigated potential genotype-dependent receptor-ligand interactions across multiple cell types using CellPhoneDB (23). We found pervasive genotype-dependent differences in the number of receptor-ligand interactions, starting as early as P15 with changes between fibroblasts and epithelial cells followed by an expansion into other cell type pairs at 8 months of age (Figure 5A and Supplemental Figure 7). For the CD8TEs, the greatest genotypic difference was observed from their interaction with fibroblasts compared with other cell types at 8 months of age (Figure 5A), suggesting fibroblasts as one of the major cell types to modulate the activation of CD8TEs. We then examined the specific receptor-ligand pairs that differed between CD8^+^ T cells and fibroblasts in *Hhip^+/–^* mice as compared with *Hhip^+/+^* mice. IFN-γ and IFN-γ receptor interaction between CD8TTEs and fibroblasts was significantly different in *Hhip^+/–^* mice at as early as 4 months of age (Figure 5B), accompanied by IFN-γ–induced cytokine-receptor interactions, such as CXCR6-CXCL16 (24). In contrast, such associations were absent for *Hhip^+/+^* mice until 11 months of age, indicative of accelerated inflammation in *Hhip^+/–^* mice due to fibroblasts-CD8^+^ T cells communications at the molecular levels starting as early as 4 months of age, preceding pathological changes (13). On the other hand, Th2-induced cytokine interactions, such as CCR2-CCL11 and TSLP-TSLPR, were absent in *Hhip^+/–^* mice, supporting the shift of CD8^+^ T cells to IFN-γ–producing type 1 CD8^+^ T cells in *Hhip^+/–^* mice (Figure 5B, bottom).

### Fibroblast-derived IL-18 enhances IFN-γ production in CD8^+^ T cells from Hhip^+/–^ mice.

**Figure 6:**
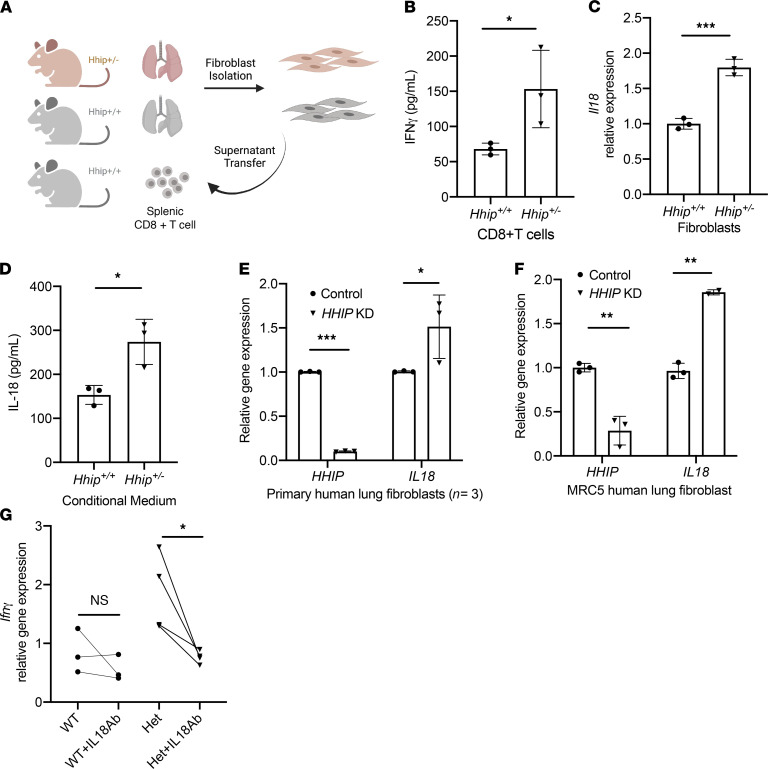
*Hhip^+/–^* lung fibroblasts induce increased IFN-γ production from CD8^+^ T cells. (**A**) Experimental design. Splenic CD8^+^ T cells from *Hhip^+/+^* mice were treated with conditioned medium (CM) from lung fibroblasts from 11-month-old *Hhip^+/+^* and *Hhip^+/–^* mice for 24 hours. (**B**) WT CD8^+^ T cells incubated with *Hhip^+/–^*-derived CM produced increased levels of IFN-γ. (**C**) Lung fibroblasts from *Hhip^+/–^* mice have higher expression of *Il18*. (**D**) IL-18 level is elevated in the *Hhip^+/–^*-derived CM measured by ELISA. (**E**) Knockdown of *HHIP* by siRNA in adult primary human lung fibroblasts (*n* = 3) and (**F**) human fetal lung fibroblast cell line MRC5 led to increased expression of *Il18*. (**G**) Expression of *Ifnγ* in WT splenic CD8^+^ T cells treated with conditioned medium derived from *Hhip^+/+^* or *Hhip+/*^–^ fibroblasts for 24 hours, with or without IL-18 neutralizing Ab (30 μg/mL) as measured by qPCR with Cd8a as the reference gene. Each point represents an individual biological replicate. Data are represented as mean ± SD, with *n* ≥ 3 per group. *P < 0.05, **P < 0.01, ***P < 0.001, unpaired Student’s *t* test (**B**–**F**), paired Student’s *t* test (**G**). Representative results are shown (from ≥2 replicated experiments with 2–4 mice in each repeat for **B**–**D** and **F**). KD, knockdown.

The single-cell RNA-sequencing analysis suggested that *Hhip^+/–^* lung fibroblasts may interact with and activate CD8^+^ T cells. As genes in the IL-18 production pathway were upregulated in *Hhip^+/–^* fibroblasts, and IL-18 is a major inducer of IFN-γ production in the absence of stimulation of the T cell antigen receptor in CD8^+^ T cells (25, 26), we hypothesized that secreted factors such as IL-18 from fibroblasts may activate CD8^+^ T cells. To test this hypothesis, splenic CD8^+^ T cells from 2-month-old WT mice were isolated and treated with conditioned medium (CM) from lung fibroblasts from 11-month-old *Hhip^+/+^* or *Hhip^+/–^* mice (Figure 6A). After 24 hours of treatment, increased expression of IFN-γ was detected in CD8^+^ T cells cultured with CM collected from *Hhip^+/–^* lung fibroblasts (Figure 6B), suggesting that *Hhip^+/–^* lung fibroblast-derived secreted factors are sufficient to drive IFN-γ production in CD8^+^ T cells. Indeed, we found significantly increased Il18 gene expression in *Hhip^+/–^* lung fibroblasts and increased IL-18 protein levels from *Hhip^+/–^* fibroblast-derived CM (Figure 6, C and D). In contrast, there was no genotypic difference in the expression of Il12a, which is also known to induce IFN-γ in T cells (Supplemental Figure 8A). Although IL-18 is highly expressed in alveolar macrophages in the lung (19), we found minimal differences in IL-18 expression in alveolar macrophages from *Hhip^+/–^* and *Hhip^+/+^* mice (Supplemental Figure 8B), suggesting that IL-18–derived activation of T cells in *Hhip^+/–^* lungs is more likely from lung fibroblasts than from macrophages.

Furthermore, knockdown of *HHIP* with siRNA led to increased expression of *IL18* in primary normal human lung fibroblasts and MRC5 lung fibroblast cell line (Figure 6, E and F). IL-18 neutralizing antibody abolished increased expression of *Ifnγ* in *Hhip^+/–^* fibroblasts (Figure 6G). These results suggest that HHIP represses IL-18 signaling and that lung fibroblast–derived IL-18, at least partially, contributes to the activation of CD8^+^ T cells, indicated by increased IFN-γ expression.

### IL-18 is increased in COPD patients carrying an HHIP risk variant.

To determine the relevance of murine findings in patients with COPD, we analyzed the relationship between genotype at the *HHIP* GWAS locus and blood cytokine profiles in a subset of non-Hispanic White patients from the COPDGene Study (*n* = 590) (Table 3) (27). *HHIP* risk variant rs1032296 (T) was significantly associated with higher IL-18 levels in serum (log transformed) by multivariable analysis (Table 4). However, no genotypic association with IFN-γ was found, possibly reflecting a more complex regulation of IFN-γ in blood samples of patients with COPD (28, 29).

## Discussion

In a human COPD study, we found that the *HHIP* genetic variant is associated with exacerbation frequency, a key phenotype associated with active inflammation (30). Single-cell RNA sequencing in lungs from the *Hhip*-haploinsufficient mouse model, which exhibits a similar inflammatory phenotype as seen in patients with COPD even in the absence of smoke exposure, revealed progressive activation of CD8^+^ T cells with age and increased expression of IFN-γ preceding emphysematous changes in *Hhip^+/–^* lungs. *Hhip* is specifically expressed in lung fibroblasts while absent in CD8^+^ T cells. Furthermore, fibroblast-CD8^+^ T cell interaction and IFN-γ–inducing pathways are enriched in *Hhip^+/–^* fibroblasts, suggesting that Hhip-expressing fibroblasts may repress CD8^+^ T cell activation, possibly through IL-18, as one of potential mediators. Consistently, the COPD risk allele of the *HHIP* genetic variant is associated with increased IL-18 levels in patients with COPD.

Among the activated forms of CD8^+^ T cells, we found a specific increase in KLRG1^+^CD8^+^ T effector cells in *Hhip^+/–^* mouse lungs. KLRG1^+^ CD8TTEs are frequently expanded in response to viral infections and produce IFN-γ (31, 32) but are also found in patients with autoimmune disease and cancer as dysfunctional T cells in persistent inflammatory conditions (33).

Both IFN-γ and IL-18 signaling pathways are known to contribute to human COPD pathogenesis. First, numbers of IFN-γ–producing T cells are increased in the lungs of patients with COPD (34, 35), and expression of IFN-γ by lung CD8^+^ T cells also correlates with COPD severity (35). Interestingly, IFN-γ recruits inflammatory cells into COPD lungs through upregulation of CXCR3 (28), which also showed increased expression along with CXCL10 in *Hhip^+/–^* lungs at 10 months of age (13). Second, IFN-γ induces the release of matrix metalloproteinase-12 (MMP-12) in a transgenic mouse model, leading to inflammation and emphysema (36, 37). Indeed, MMP-12 levels are increased in *Hhip^+/–^* lungs when lung lymphoid aggregates are evident (13). Additionally, IFN-γ synergistically acts with TNF-α to induce expression of CXCL10 via NF-κB (38), reinforcing the recruitment of CXCR3/IFN-γ–expressing T cells to the lung (39). Indeed, TNF-α signaling via NF-κB pathway was enriched in upregulated genes in *Hhip^+/–^* lungs (Supplemental Figure 6). Similarly, IL-18, a strong inducer of IFN-γ, not only promotes emphysema development in murine models (37, 40) but also functions as a biomarker and a master cytokine driving COPD progression (41). IL-18 is elevated in COPD patients’ lung and sputum samples, which correlates with IFN-γ levels (42). Consistent with our findings, stimulation of human lung CD8^+^ T cells from COPD patients in vitro with IL-18 (plus IL-12) increases IFN-γ production, and the expression of IL-18 receptor correlates with spirometrically defined COPD severity (43). Thus, our findings provide one possible mechanism to explain the genetic heterogeneity of augmented inflammation seen in COPD patients.

Whether *Hhip* regulates IL-18 expression in fibroblasts through its inhibition of the hedgehog pathway requires further study. Overexpression of the hedgehog effector *Smoothened* in lung mesenchymal cells leads to negligible inflammatory phenotypes in lungs despite evident emphysema (44), suggesting minimal impact of intrinsic hedgehog signaling in lung fibroblasts on the activation of lymphocytes (18, 19, 45). Therefore, the molecular mechanism by which HHIP represses IL-18 expression in lung fibroblasts needs future investigation.

Frequent cell-cell communication in lungs, including crosstalk between *Hhip*-expressing fibroblasts and CD8^+^ T cells, may contribute to the formation of dysfunctional T cells important for COPD pathogenesis. As shown in Supplemental Figure 7, fibroblasts also had differential receptor-ligand interactions with other cell types, including endothelial cells and ciliated epithelial cells, that were significant as early as P15. Future studies including endothelial and epithelial cells and other lung cell types interacting with fibroblasts will reveal more comprehensive functions of *HHIP* in lungs through cell-cell communications.

Aside from the contribution of the IL-18/IFN-γ axis to such fibroblast-lymphocyte crosstalk, roles of other fibroblast-derived factors need to be assessed in comprehensive cytokine screenings. Furthermore, the association of HHIP genotype with serum IL-18 levels may not be completely attributable to regulation of HHIP on IL-18 expression in lung fibroblasts, which requires further studies. Furthermore, future studies on blocking the lymphocyte inflammation or IL-18/IFN-γ signaling to ameliorate the emphysema phenotype in *Hhip^+/–^* murine models and examination of expression of IL-18 in human lung tissue are also warranted to pinpoint the therapeutic applications.

Notably, the murine Hhip haploinsufficiency model displays an array of inflammatory phenotypes that resembles human COPD patients, including the peri-airway location of lymphoid aggregates (12), presence of increased CD8^+^ T cells in lymphoid aggregates, and increased number of activated CD8^+^ T cells (12, 13, 43). The location of these specific inflammatory features coincides with the peribronchial location of Hhip-expressing murine fibroblasts as shown by Tsukui and colleagues (46), and the observation of IL-18 induction in human adult lung fibroblasts upon silencing of HHIP suggests a plausible role of the mesenchymal HHIP in the inflammatory pathophysiology of COPD. However, recent single-cell RNA sequencing expression studies in adult human lungs suggest additional alveolar expression of HHIP (46–48). Specifically, although we find that HHIP expression is restricted in the fibroblast population in mouse lungs (Figure 1, D and E), as shown in other publications (19, 46, 48), as well as neonatal human lungs (49), strong HHIP expression was also found in a subset of type 2 alveolar epithelial cells in adult rat, pig, and human lungs (47). These observations raise questions such as the long-term effect of HHIP expression in the developing lung mesenchyme, and the role of HHIP in type 2 alveolar epithelial cells to the airway inflammation, which requires further investigation in future studies. Taken together, further studies with conditional ablation of HHIP in adult tissues and cell type–specific ablation of HHIP in human lung organoid models or species with both epithelial and mesenchymal expression of HHIP, such as rats, would elucidate the developmental, compartment-specific effect of HHIP in COPD pathogenesis.

In sum, our study provides the utility of single-cell transcriptomics in inferring cell type–specific gene expression and intercellular interactions and suggests a potential mechanism by which the genetic factor *HHIP* may control lymphocytic inflammation in COPD. Additionally, receptor-ligand analysis using enriched single-cell RNA-sequencing data provided cell-cell communication roadmaps in murine lungs from mice at 4 different ages. COPD exacerbations are treated with antiinflammatory, immunomodulatory drugs, such as azithromycin (50), with incompletely defined mechanism of actions. With comprehensive gene signatures in multiple immune cell types in lungs associated with age-dependent emphysema, our work may ultimately facilitate the development of novel immunomodulatory therapies in COPD.

## Methods

### Human patients

Details of the COPDGene Study (Genetic Epidemiology of COPD, ClinicalTrials.gov NCT00608764) have been described previously (27). The COPDGene Study enrolled 10,192 smokers with and without COPD between November 2007 and April 2010. Patients were clinically stable, with at least 30 days since their last exacerbation. Of the 10,300 participants (including 108 lifelong nonsmokers), 6884 (67%) were non-Hispanic White (NHW) participants, of whom 6646 (97%) had genotype data available for *HHIP* variant rs1032296. We analyzed 6627 NHW patients with lung function information and a subset of 3405 patients with COPD defined as the postbronchodilator ratio of FEV_1_/FVC < 0.7. Genotyping was performed using HumanOmniExpress array (Illumina) (2). Blood cytokines were measured in a subset of patients (*n* = 590) as previously published (51). Briefly, blood biomarkers were measured by customized 13-panel multiplex assays (Myriad-RBM) (51). Measurement results for IL-18 and IFN-γ were used for analysis.

### Mice

*Hhip^+/–^* mice, as described previously, were generated by replacing exon 1 of *Hhip* with a lacZ reporter gene (52) followed by backcrossing to C57BL/6J background (11). *Hhip^+/–^* mice and littermate WT *Hhip^+/+^*, a total of 18, with matching age and sex were harvested at different ages for single-cell RNA sequencing and in vitro experiments.

### Generation of lung single-cell suspension

For harvesting, mice were euthanized by CO_2_ asphyxiation followed by exsanguination. Lungs were perfused with chilled PBS via the right atrium. For lung cells’ isolation without enrichment (15 out of 18 samples), dispase (50 U/mL) was instilled into lungs, which were dissected and minced on ice into approximately 1 mm^3^ cubes. Lungs were digested using collagenase/dispase for 30 minutes at 37°C, then passed through 70 μm and 40 μm cell strainers. Cells were centrifuged at 300 RCF for 5 minutes, then incubated with RBC lysis buffer. Cells were stained with trypan blue for viability assessment, which showed greater than 85% viability with this method. The single-cell suspension was sorted by FACS for DAPI-negative viable cells using MoFlo Astrios EQ (Beckman Coulter).

For enriched CD45-negative lung cells (two 4-month samples and one 8-month sample), lungs were digested using a mouse lung isolation kit with gentleMACS dissociator (Miltenyi Biotec), then were passed through 70 μm and 40 μm cell strainers. After centrifugation at 300 RCF for 5 minutes, cells were incubated with RBC lysis buffer. Cells were then stained with DAPI, DRAQ5 (Abcam), and CD45-PE Ab (BioLegend) and selected for DAPI-negative, DRAQ5-positive, and CD45-negative cells using On-chip Sort cell sorter (On-chip Biotechnologies) (53).

### Single-cell RNA sequencing

#### Single-cell RNA sequencing using 10x Chromium.

Lung single-cell suspension was loaded onto a 10x Chromium Single Cell instrument per manufacturer’s instructions. cDNA amplification and library construction were performed according to the 10x Chromium Single Cell 3′ v2 manufacturer’s protocol. Quality control of the library was performed by Agilent Bioanalyzer and qPCR by using an Illumina Library Quantification Kit (KAPA Biosystems, KK4824). Libraries were sequenced on the Illumina HiSeq (Harvard Biopolymers Facility) or NovaSeq (Novogene) platform, using paired-end reads, with the following read length: 26 bp read 1 for cell barcode and UMI, 8 bp i7 index for sample index, and 98 bp for transcript.

#### Single-cell RNA sequencing using inDrops.

For 8-month-old mouse samples, the single-cell suspension was directly loaded into a microfluidic device. Cells were encapsulated with barcoded hydrogel microspheres, reverse transcriptase, and lysis reagents. Primers were photo-cleaved by UV exposure after encapsulation as previously described (54). cDNA amplification and library construction (v3) were performed at the Single Cell Sequencing Core at Harvard Medical School. Indexed libraries were pooled and sequenced on an Illumina NextSeq 500 (Harvard Bauer Facility), using paired-end reads, with the following read length: 61 bp for transcript, 14 bp for barcode and UMI, 8 bp i7 index for part of barcode, and 8 bp i5 index for sample index.

### Cell culture studies

#### Cell line.

Human fetal lung fibroblast cell line MRC5 (ATCC, CCL-171) was cultured in DMEM supplemented with 10% fetal bovine serum (FBS) and antibiotics in a humidified incubator at 37°C with 5% CO_2_.

#### Primary cell isolation and culture.

Primary mouse lung fibroblasts were isolated by culturing the enzymatically digested lungs with liberase (MilliporeSigma) in DMEM/F12 containing 15% FBS in a humidified incubator at 37°C and 5% CO_2_. Fibroblasts were passaged and cultured in DMEM supplemented with 10% FBS before being used for experiments. Cells were used between passages 1 and 2.

Primary mouse alveolar type 2 epithelial cells were isolated as previously described (55); briefly, lung was enzymatically digested with dispase. Macrophages were removed by incubation with biotinylated anti-CD45, anti–TER-119, and anti-CD16/32 antibodies (clones 30-F11, TER-119, and 2.4G2, BD Biosciences) and then subjected to magnetic selection with streptavidin-conjugated magnetic beads (Invitrogen). Cells were seeded onto Petri dishes precoated with mouse IgG (I5381, MilliporeSigma) and cultured for 2 hours at 37°C. Nonadherent alveolar epithelial cells were removed from IgG plates and resuspended in DMEM/F-12 medium supplemented with 10% FBS.

CD8^+^ T lymphocytes from mouse lungs and spleens were isolated by negative selection using CD8a^+^ T Cell Isolation Kit (Miltenyi Biotec).

Alveolar macrophages were isolated by performing bronchoalveolar lavage (total 10 mL), and purity of alveolar macrophage (>90%) was confirmed by Diff Quick staining (MilliporeSigma) after cytospin preparation.

Splenocytes were prepared by excising the spleen and passing the cells into a 40 μm cell strainer.

Primary human lung fibroblasts were isolated from the lung tissues of healthy individuals (Marsico Lung Institute, University of North Carolina at Chapel Hill, North Carolina). The lung tissue was cut into small pieces and seeded onto culture dishes at 37°C in a humidified 5% CO_2_/air incubator. The dishes were placed upside down for 2 hours, then turned back, and DMEM was added. The primary human lung fibroblasts grew to 90% confluence within 2 to 3 weeks for subsequent experiments.

#### Immunofluorescence staining and flow cytometry.

Lung CD8^+^ T cells were washed in PBS containing 0.5% bovine serum albumin and 2 mM EDTA, then incubated with the following antibodies: CD127 (A7R34) PE (eBioscience 12-1271-81), Klrg1 (2F1) APC (eBioscience 17-5893-81), CD4 (RM4-5) PerCP-Cy5.5 (eBioscience 45-0042-82), Tcrβ BV605 (BioLegend 109241), CD45.2 BV510 (BioLegend 109837), and Cd8a BV711 (BioLegend 100747). Measurements were performed on a FACSymphony flow cytometer (BD), and analysis was performed with FlowJo software.

#### Fibroblast-derived supernatant transfer.

On day 1, fibroblasts from 11-month-old mice were plated in 12-well culture plates at 5 × 10^5^ cells/well in DMEM with 10% FBS. On day 2, 1 × 10^6^ fresh splenic CD8^+^ T cells harvested from 2-month-old *Hhip^+/+^* mice were cultured in supernatant collected from *Hhip^+/+^* or *Hhip^+/–^* fibroblasts with the addition of anti-CD3 and anti-CD28 Ab (0.25 μg/mL each, 562163, 553294, BD Biosciences). CD8^+^ T cells were maintained at 37°C with 5% CO_2_ for an additional 24 hours before subsequent qPCR analysis. In experiments for Figure 6G, IL-18 neutralizing Ab (30 μg/mL, D048-3, R&D Systems) was added to CD8^+^ T cells for 24 hours on day 2 before cell collection for quantitative real-time PCR analysis.

#### RNA interference.

We transfected siRNAs targeting human *HHIP* and nontargeting control pools (J-013018-09-0010, D-001810-10-05, respectively, Dharmacon) into primary human lung fibroblasts using Lipofectamine RNAi/MAX (Invitrogen). The culture medium was changed to a normal medium 2 hours after transfection.

### Quantitative reverse transcription PCR

Total RNA from cells was isolated with RNeasy Mini Kit (QIAGEN) according to the manufacturer’s protocol. A total of 100 ng of RNA was reverse-transcribed to cDNA with High-Capacity cDNA Reverse Transcription Kit (Life Technologies). For qPCR, TaqMan gene expression assays were used on a QuantStudio 12K Real-Time PCR system (Applied Biosystems). FAM-conjugated TaqMan probes for *Hhip*, *Ifnγ*, *Il18*, *Il12a*, *Il12b*, *Gapdh*, *Cd8a*, and *Tbp* (mouse) and *HHIP*, *IL18*, and *GAPDH* (human) (IDT) were used with TaqMan Gene Expression Master Mix (Applied Biosystems). Gene expression values were normalized to *GAPDH* for isolated fibroblasts and MRC5 cell lines and to *Cd8a* and *Tbp* for isolated CD8^+^ T lymphocytes and macrophages. Expression levels of target genes were calculated based on the 2^-ΔΔCt^ method. For undetermined Ct values, Ct value of 40 was used to calculate relative expression level.

### ELISA

Supernatant from mouse or human fibroblasts was analyzed for IL-18 levels using the Mouse IL-18 Platinum ELISA (Invitrogen, BMS618-3) or Human Total IL-18/IL-1F4 Quantikine ELISA Kit (R&D Systems). IFN-γ levels in cell culture media from CD8^+^ T lymphocytes were analyzed using Mouse IFN-γ ELISA MAX Standard (BioLegend) according to the manufacturer’s protocol.

### Single cell RNA-sequencing data analysis

#### 10x data preprocessing.

Sequencing output was demultiplexed by the Cell Ranger (version 2.0.0) pipeline. The number of cells in each sample was estimated by the Cell Ranger software (Estimated Number of Cells). LacZ transgene mapping was performed by incorporating LacZ sequence to the mm10 reference genome to confirm the genotypic identity of *Hhip^+/–^* mice.

#### inDrops data preprocessing.

Sequencing output was demultiplexed by the inDrops pipeline. To exclude potential doublets and empty cells, we filtered cells with UMI counts over 3000 or less than 200, as well as cells with mitochondrial percentage over 15% (Supplemental Table 1). After constructing the raw count matrix, we used the R package Seurat (v3) (56) for data analysis.

#### Seurat.

Library count matrices were processed with variance stabilizing transformation (*SCTransform* workflow) in Seurat v3.1.4. The fraction of mitochondrial UMI counts per cell were regressed out. Utilizing the Pearson residuals by the variance stabilizing transformation of libraries, all library count matrices were integrated using a reference library (4 m Het-2) that had the highest sequencing depth per cell. The reference-based integration did not differ in a biologically meaningful fashion from the full pairwise comparison suggested by the standard workflow. For the anchor finding and integration, 30 dimensions were used. Compared with simple merge of count matrices, batch effect was mainly driven by cell capture technology (inDrops vs. 10x), sequencing platform and/or preparation (HiSeq, NextSeq vs. NovaSeq; whole lung cells vs. On-chip–enriched CD45-negative cells). This batch effect was effectively removed after the integration procedure was assessed by the merge of clusters from 3 batches in the UMAP embedding plot.

The integrated Pearson residual matrix was used to cluster cells. Specifically, principal component analysis generated 40 principal components that were subsequently used for neighborhood graph construction, UMAP embedding, and cluster identification (Leiden algorithm, algorithm = 4 for *FindClusters* function in Seurat v3.1.4). The choice of the number of principal components (PCs) was determined partially by the SD of each PC as assessed by *Elbowplot* function in Seurat v3.1.4. The overall embedding and clustering were not sensitive by the choice of PC numbers. To cluster all cells, we used a resolution parameter of 1.5. For T cell and fibroblast population, subclustering was necessary to identify detailed subtypes (NKT cells and fibroblast subtypes), as increases in PCs or resolution parameters were ineffective in distinguishing subtle differences.

To annotate cluster identities objectively, published single-cell data annotation from a mouse cell atlas (18) were transferred to our data for annotation of lung structural cell types using *TransferData* function in Seurat v3.1.4. Subsequently, the graph-based clusters from the Leiden algorithm were subjected to marker identification by Wilcoxon rank sum test using the wilcoxauc function of the presto package (1.0.0) (57), utilizing the Pearson residuals from the variance stabilizing transformation of the Seurat workflow. Together with the initial label from the mouse atlas, the markers were manually inspected to assign the overall annotation for each graph-based cluster. For downstream analysis, several graph-based clusters were merged with a broader term (dendritic cells, B cells, endothelial cells, and alveolar epithelial cells), in particular if they were shown as a continuum in the UMAP embedding plot. The T cell cluster (*Cd3a*^+^ cell population) and NK and ILC populations that formed a continuum in the UMAP embedding plot were subjected to further marker identification by pairwise comparison of adjacent clusters and subtype identification by the ImmGen DataBrowser (Gene Skyline, ImmGen ULI RNASeq) (58), to compare for specific markers for reference populations. Final main clustering resulted in 26 cell (sub)types from 44 clusters (resolution = 1.5). Note that a few PCs were enriched for cell cycle genes, and 439 cells were grouped mainly based on these PCs and estimated to be in G2M and S phase based on the *CellCycleScoring* function in Seurat v3.1.4. Neither removing the cell cycle gene–enriched PC, nor regressing out the scores of S phase and G2M phase derived from the *CellCycleScoring* function, could merge the cells into original cell type clusters, suggesting that these cells had insufficient information to be incorporated into the respective cell type clusters. Given that these cells did not have obvious bias in terms of genotype and age to the entire population, and the majority of S and G2M phase estimated cells were still found in cell type clusters, no further attempt to reassign the 439 cells (annotated as “Dividing”) into subtype annotation was made.

In the *Cd3a*^+^ cell population group, NKT cell population was not clearly assigned as a graph-based subcluster, likely because of the very close transcriptomic profile to related cell clusters, suggested by the close overlap of scores from the R package singleR (20) with ImmGen reference database (58). Thus, the T cell, NK, and ILC cluster was subsetted. To minimize batch effect from technical platform and cell capture method, we grouped the cells based on batches and performed the integration procedure after variance stabilizing transformation was conducted. Subclustering was performed using 20 PCs with Leiden algorithm with resolution of 0.8.

For fibroblast subclustering, we conducted the same approach as described above by grouping the subsetted libraries into batches (inDrops, CD45-negative enriched 10x, whole lung 10x) and integration. Subclustering was performed using 10 PCs with Leiden algorithm with resolution of 0.8. We compared the individual fibroblast subclusters with published data (Gene Expression Omnibus, GEO: GSE104154) (19) using *TransferData* function in Seurat v3.1.4. Before the label transfer, the original count matrix from the Xie et al. data set (21) was processed in a similar fashion, using variance stabilizing transform and 40 PCs. The annotation matched well with the newly processed cluster and embedding diagram. Transferred labels also matched well to the graph-based clusters. Note that for the myofibroblast label, a distinct, small smooth muscle cell cluster was also labeled, given that the Xie et al. data set did not contain this cell type.

Cell type–specific differential gene expression analysis was performed on Pearson residuals from the *SCTransform* in Seurat (59). We applied Wilcoxon rank sum test from presto package; differential expression was defined as log fold change > 0.2 and adjusted *P* (FDR corrected) < 0.05.

Average expression was calculated by natural log transformation of averaged expression of RNA assay using AverageExpression function in Seurat.

#### Receptor-ligand interaction.

Receptor-ligand interaction analysis was performed by CellPhone DB python package (23). Orthologous human gene names replaced mouse gene symbols using the bioMart R package (60). Counts from nonorthologous genes were discarded prior to analysis. The normalized count data originally processed in the total UMI counts were used for the analysis. The data were split into genotype and age for individual processing, and 1000 iterations were used for *P* value calculation. Statistically significant interactions between cell types for each age/genotype group were compared, and the number of interactions that differed between genotypes were counted and shown in the heatmap from mice at each age. Statistically significant interactions among cell types with genotypic differences from mice at each age were shown as receptor-ligand pairs in a dot plot.

#### Functional enrichment analysis.

GO term enrichment was performed using g:Profiler (61).

#### Data availability.

Genomic data for the manuscript are available from GEO GSE149843 (https://www.ncbi.nlm.nih.gov/geo/query/acc.cgi?acc=GSE149843).

### Statistics

Nongenomic experimental data were analyzed using a 2-tailed Student’s unpaired *t* test unless otherwise stated, using GraphPad Prism software. Student’s *t* test with Welch’s correction was performed when group sizes were not equal. *P* < 0.05 was considered significant.

#### Human genotype analysis.

Genetic analysis of human patients was performed using additive genetic models. Negative binomial regression was used to assess *HHIP* genotype association with COPD exacerbation frequency in the year prior to enrollment adjusted for genetic ancestry PCs, age, sex, baseline St. George’s Respiratory Questionnaire score, FEV_1_ percent predicted, and gastroesophageal reflux disease.

Linear regression was used to evaluate *HHIP* genotype association with log-transformed IL-18 and IFN-γ adjusted for genetic ancestry PCs, age, sex, and current smoking status. All analyses were performed using R. All *P* values were 2 sided, and less than 0.05 was considered statistically significant.

### Study approval

All animal studies were approved by the Institutional Animal Care and Use Committee of Brigham and Women’s Hospital.

COPDGene (ClinicalTrials.gov NCT00608764) is a multicenter, observational study (27). Institutional review board approval was obtained at each of the 21 participating clinical centers, and written informed consent was obtained from all participants.

## Author contributions

JHY and XZ designed the study. JHY performed single-cell RNA sequencing. JHY and CL performed data analysis. JHY, TL, and SL performed in vitro assays and qPCR. SX maintained mice colonies and performed genotyping. JHY, CL, TL, EYK, JLC, LP, RBP, EKS, CPH, and XZ interpreted data and edited the manuscript.

## Supplementary Material

Supplemental data

Supplemental data file 1

Supplemental data file 2

## Figures and Tables

**Table 1:**
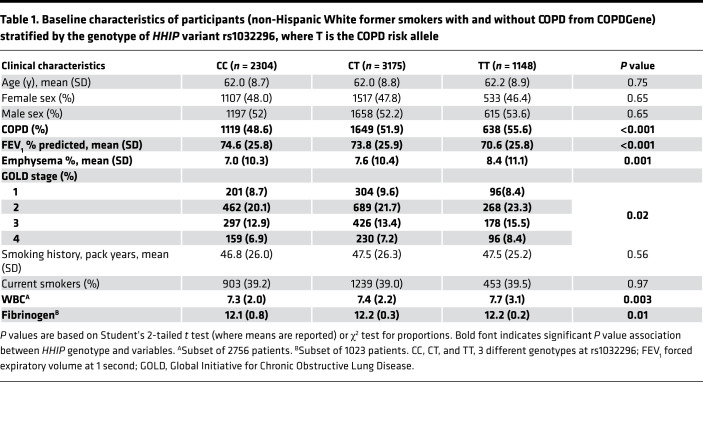
Baseline characteristics of participants (non-Hispanic White former smokers with and without COPD from COPDGene) stratified by the genotype of *HHIP* variant rs1032296, where T is the COPD risk allele

**Table 2:**
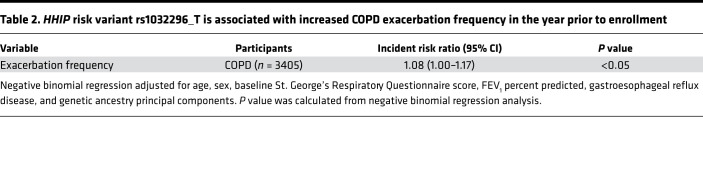
*HHIP* risk variant rs1032296_T is associated with increased COPD exacerbation frequency in the year prior to enrollment

**Table 3:**
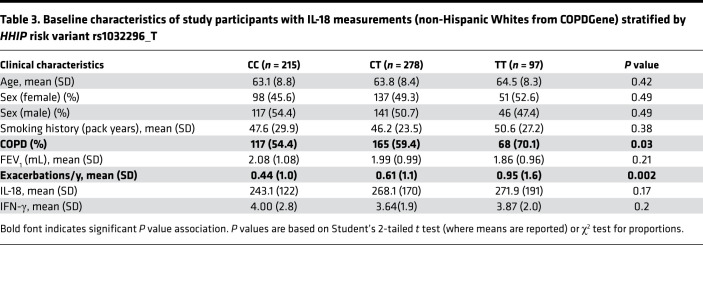
Baseline characteristics of study participants with IL-18 measurements (non-Hispanic Whites from COPDGene) stratified by *HHIP* risk variant rs1032296_T

**Table 4:**
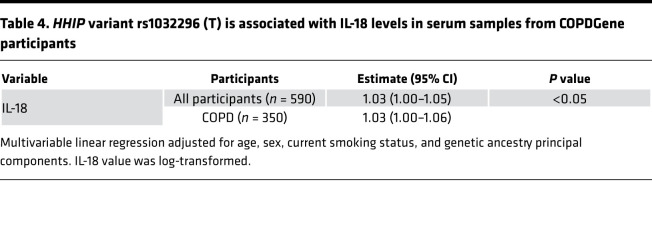
*HHIP* variant rs1032296 (T) is associated with IL-18 levels in serum samples from COPDGene participants

## Version 1 08/10/2021

In-Press Preview

## Version 2 09/08/2021

Electronic publication
