# Supplementary material for: Hedgehog interacting protein–expressing lung fibroblasts suppress lymphocytic inflammation in mice
Source: JCI Insight. 2021 Sep 8;6(17):e144575. doi: 10.1172/jci.insight.144575 (PMC8492352; doi:10.1172/jci.insight.144575)
Supplement: Supplemental data [file jciinsight-6-144575-s122.pdf]

Hedgehog interacting protein-expressing lung fibroblasts suppress lymphocytic inflammation in mice

Jeong H. Yun[1,2*], ChangHee Lee[3*], Tao Liu[1], Siqi Liu[1], Edy Kim[2], Shuang Xu[1], Jeffrey L. Curtis[4, 5], Luca Pinello[6], Russel P. Bowler[7], Edwin K. Silverman[1], Craig P. Hersh[1,2], Xiaobo Zhou[1]

SUPPLEMENTARY MATERIALS

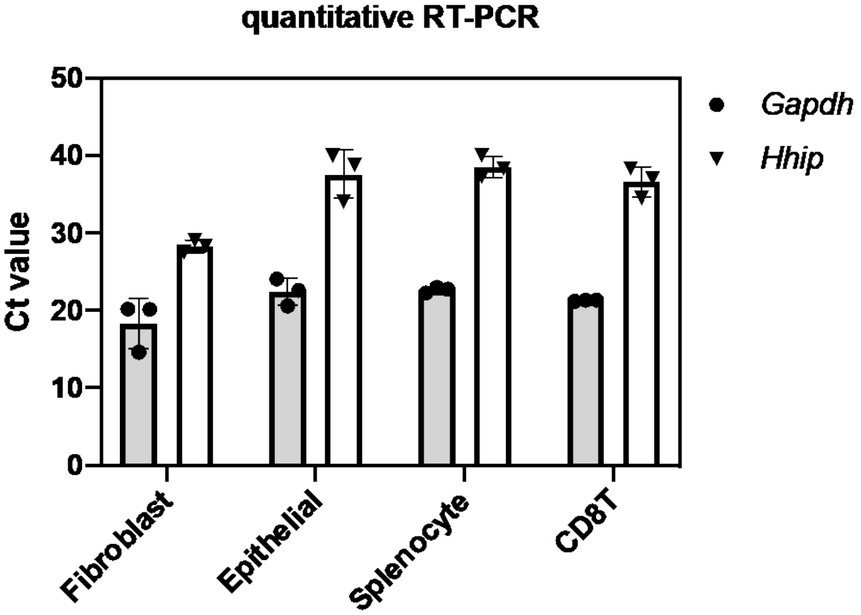

Figure S1. Cycle threshold (Ct) values of *Hhip* and housekeeping gene (*Gapdh*) from real-time PCR in various lung cell types. Each cell type was isolated by cell culture and magnetic-activated cell sorting (fibroblasts, alveolar epithelial cells and CD8+T cells) and spleen samples are from single cell suspension generated by mechanical disruption of the spleen from 4-month-old wild type C57BL6 mice (n=3). Data are represented as mean ± SD. Each point represents individual biologic replicate. Fibroblast: lung fibroblast, Epithelial: alveolar type 2 cells, CD8T: lung CD8+T cells

Genotype	Age	Rep	Sequencer	Platform	Estimated Number of Cells	Median Genes per Cell	Mean Reads per Cell	Median UMI per Cell	Valid Barcodes (%)	Reads Mapped Confidently to Exons (%)	Fraction Reads in Cells (%)	Total Genes Detected	Total number of cells analyzed
Het	P15	1	HiSeq	10x	1,260	564	53,248	1,235	95.20%	50.80%	86.1%	14,638	1,206
Het	P15	2	HiSeq	10x	377	930	182,825	2,356	96.30%	48.20%	86.0%	14,169	341
Het	4m	1	HiSeq	10x	3,004	1,060	33,802	2,521	98.30%	70.40%	91.7%	16,863	2,976
Het	4m	2	HiSeq	10x	5,107	1,081	17,650	2,567	98.30%	70.90%	93.9%	17,491	5,055
Het	4m	3	NovaSeq	10x	4,177	706	58,018	1,293	97.2%	61.3%	88.3%	16,400	4,160
Het	8m	1	NextSeq500	inDrops	1,119	426	5,330	883	44.00%	59.57%	19.2%	14,591	653
Het	8m	2	NextSeq500	inDrops	2,339	440	4,347	886	46.65%	59.35%	33.4%	15,465	899
Het	8m	3	NovaSeq	10x	6,612	825	121,309	1,648	97.2%	58.5%	88.2%	17,597	6,022
Het	11m	1	HiSeq	10x	2,315	1,142	20,826	2,623	98.30%	64.70%	92.1%	16,681	2,316
Het	11m	2	HiSeq	10x	3,362	1,013	28,663	2,275	98.20%	70.70%	92.0%	17,071	3,324
Wt	P15	1	HiSeq	10x	1,233	776	50,224	1,780	96.70%	51.10%	85.4%	14,738	1,142
Wt	P15	2	HiSeq	10x	1,054	706	75,251	1,542	96.50%	47.10%	81.8%	14,532	930
Wt	4m	1	HiSeq	10x	2,721	967	38,510	2,137	98.20%	67.50%	90.5%	16,522	2,701
Wt	4m	2	NovaSeq	10x	1,981	847	336,477	1,850	97.5%	62.9%	91.9%	15,558	1,943
Wt	8m	1	NextSeq500	inDrops	467	490	7,378	1,067	39.17%	60.79%	16.1%	12,870	311
Wt	8m	2	NextSeq500	inDrops	2,968	349	3,213	794.5	45.83%	66.53%	36.9%	14,774	824
Wt	11m	1	HiSeq	10x	1,678	1,120	26,650	2,644	98.30%	68.80%	92.7%	16,320	1,639
Wt	11m	2	HiSeq	10x	2,552	1,132	47,514	2,688	98.10%	69.70%	92.4%	16,941	2,510

Table S1. Sequencing Quality Control Metrics Summary of lung samples used in the single cell RNA-seq experiment.

Rep: replicate, p15: postnatal day 15, 4m: 4 months, 8m: 8 months, 11m: 11months, Het: *Hhip+/-*, Wt: *Hhip+/+*

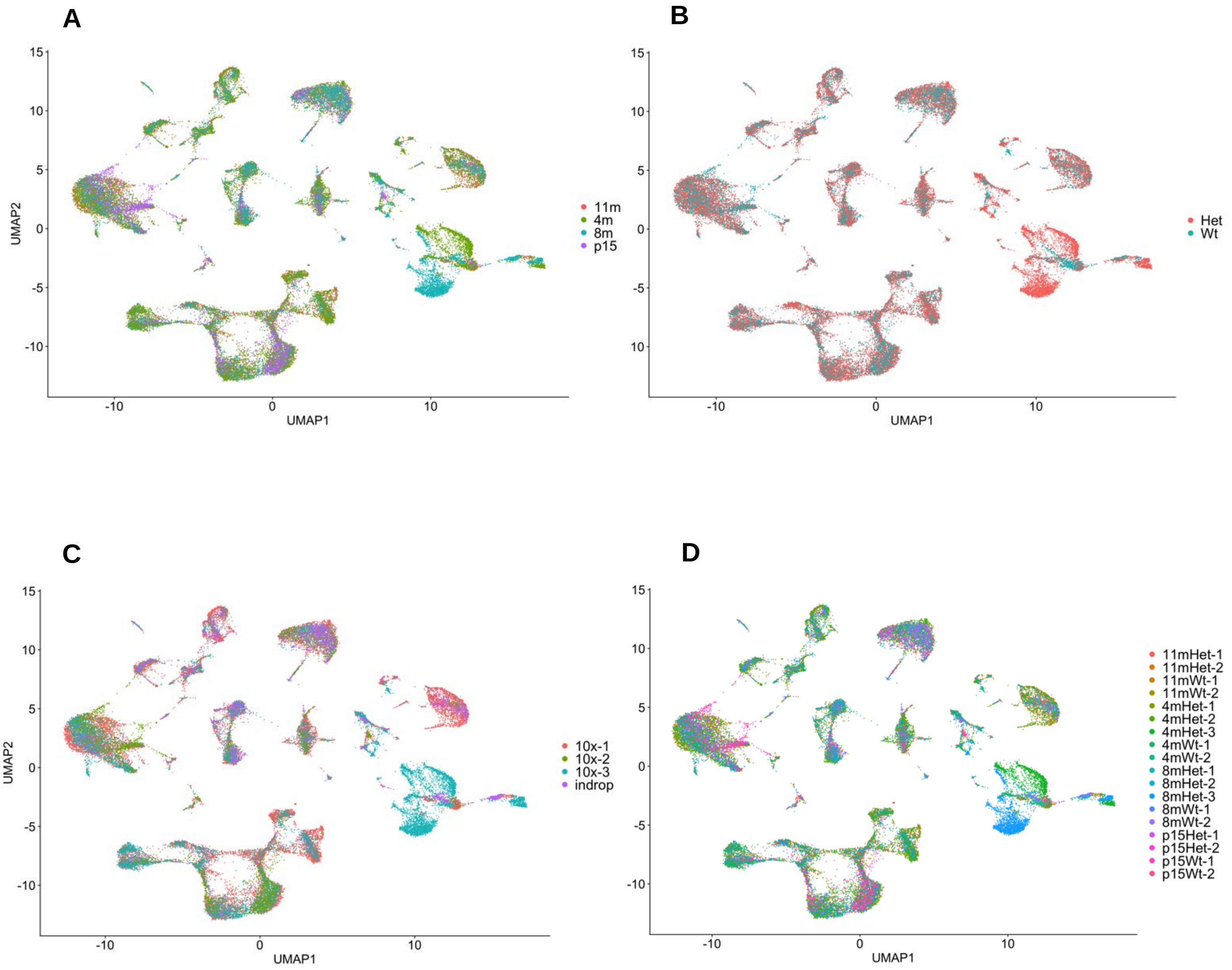

Figure S2. Uniform manifold approximation and projection (UMAP) visualization of single cell RNA sequencing of 38,875 cells. Cells were indicated by various colors based on (A) the originating age of the samples, (B) genotypes, (C) the technical batches, (D) individual libraries. 10x-3 batch was enriched for non-CD45 cells, including fibroblast and endothelial population. Cell clusters were not altered by age, *Hhip* haploinsufficiency or batch effects.

p15: postnatal day 15, 4m: 4 months, 8m: 8 months, 11m: 11months, Het: *Hhip+/-*, Wt: *Hhip+/+*

A

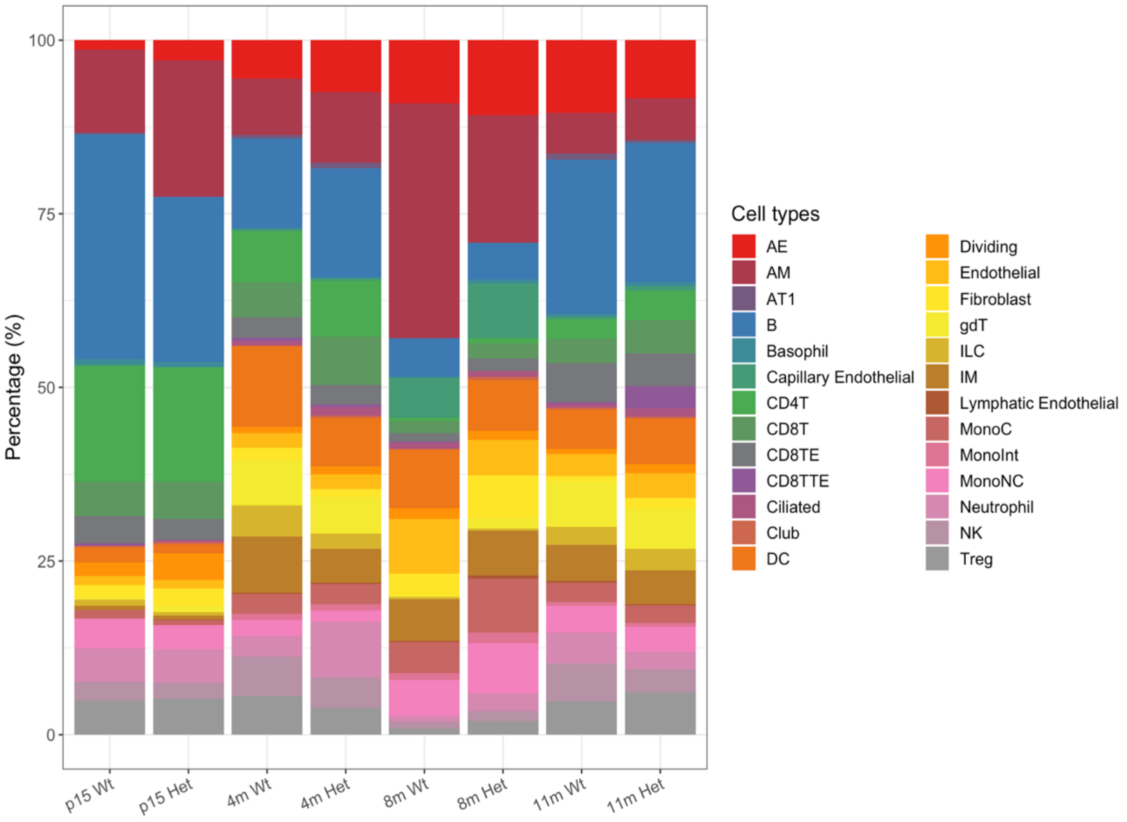
100
75
50
25
0
Percentage (%)
p15 Wt
p15 Het
4m Wt
4m Het
8m Wt
8m Het
11m Wt
11m Het
Cell types
AE
AM
AT1
B
Basophil
Capillary Endothelial
CD4T
CD8T
CD8TE
CD8TTE
Ciliated
Club
DC
Dividing
Endothelial
Fibroblast
gdT
ILC
IM
Lymphatic Endothelial
MonoC
MonoInt
MonoNC
Neutrophil
NK
Treg

B

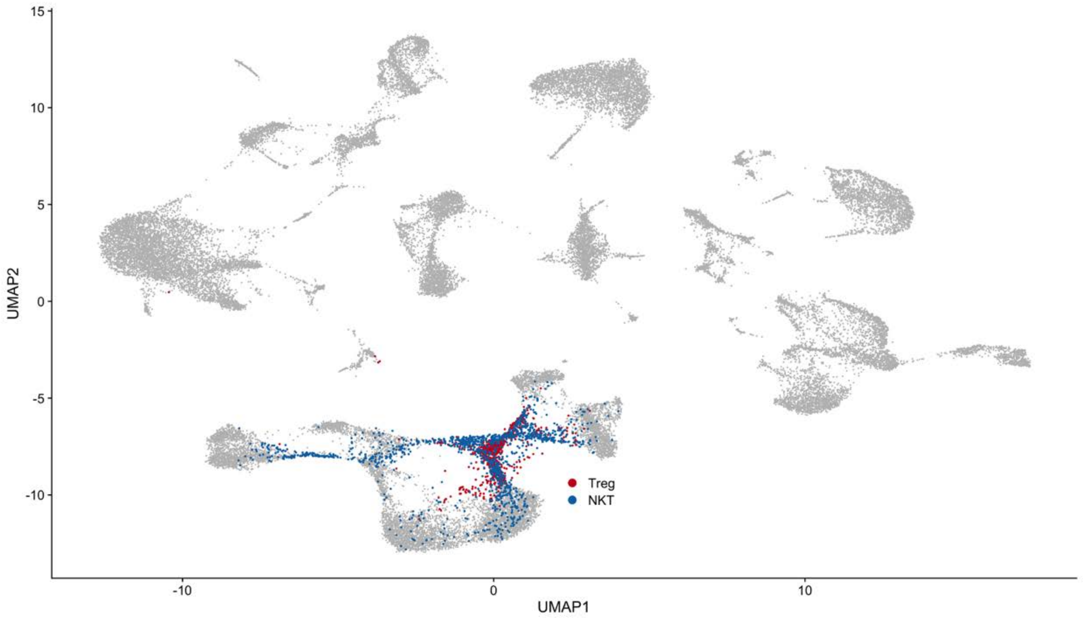
15
10
5
0
-5
-10
UMAP2
-10
0
10
UMAP1
Treg
NKT

C

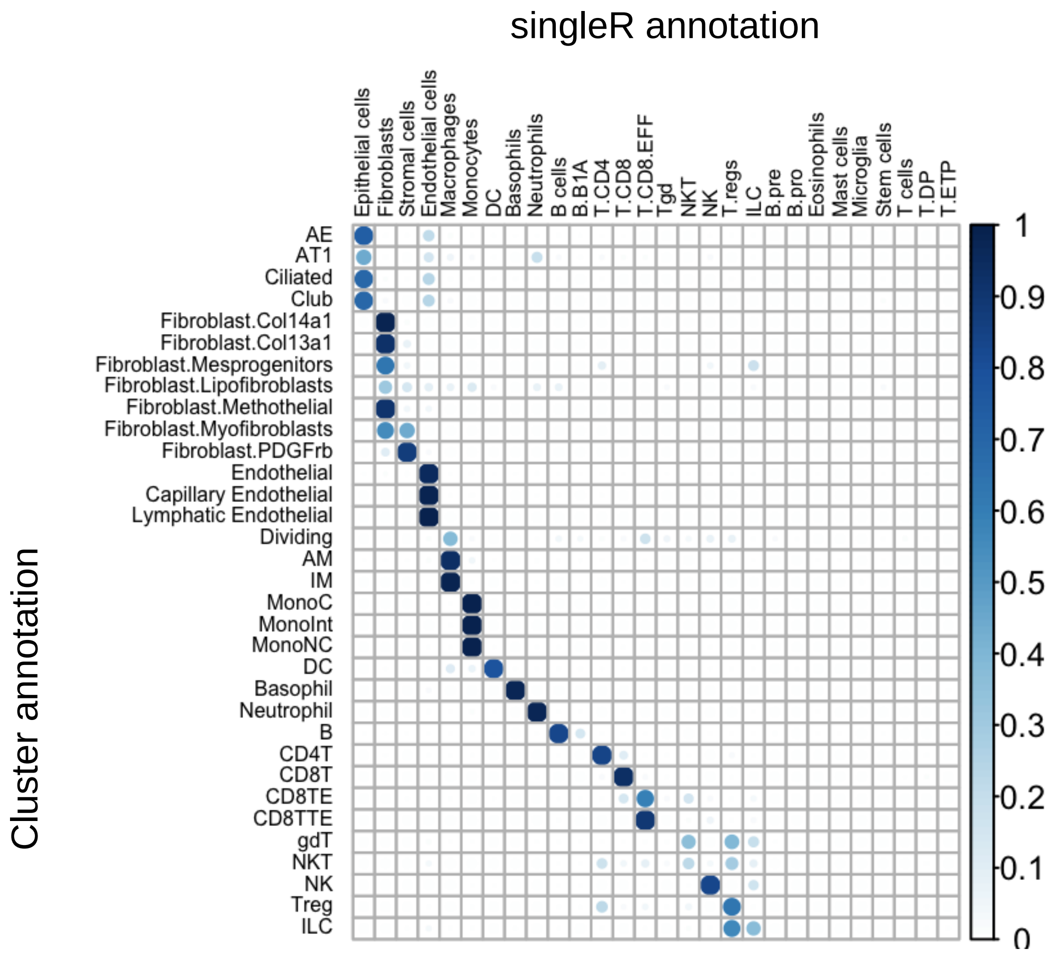

Figure S3. Cell type annotation in single cell RNA sequencing data. (A) Percentage of cells of each cell type at p15, 4 months, 8 months and 11 months of age. Samples in 10x batch 3 enriched for CD45 negative population were excluded for this analysis. Single cell RNA-sequencing was performed in different technical platform (inDrops) for 8 months samples with smaller cell numbers, suggesting technical bias affecting cellular proportions. (B) Annotation of natural killer T (NKT) cells. NKT cells (Blue) were grouped within the regulatory T cells (Treg, Red) based on graph-based clustering. (C) Dot plot comparing annotation of graph-based clusters and manual annotation of NKT cells (Cluster annotation) with annotation of cell types based on singleR program based on ImmGen data (singleR annotation)(1)

p15: postnatal day 15, 4m: 4 months, 8m: 8 months, 11m: 11 months, Wt: *Hhip+/-*, Het: *Hhip+/-*

AE: alveolar epithelial cell, AM: alveolar macrophage, AT1: type 1 alveolar epithelial cell, CD8T: CD8+T cell, CD8TE: CD8+effector memory T cell, CD8TTE: CD8+terminal effector T cell, DC: dendritic cell, gdT: gamma delta T cell, ILC: innate lymphoid cell, IM: interstitial macrophage, MonoC: classical monocyte, MonoInt: intermediate monocyte, MonoNC: non-classical monocyte, NK: natural killer

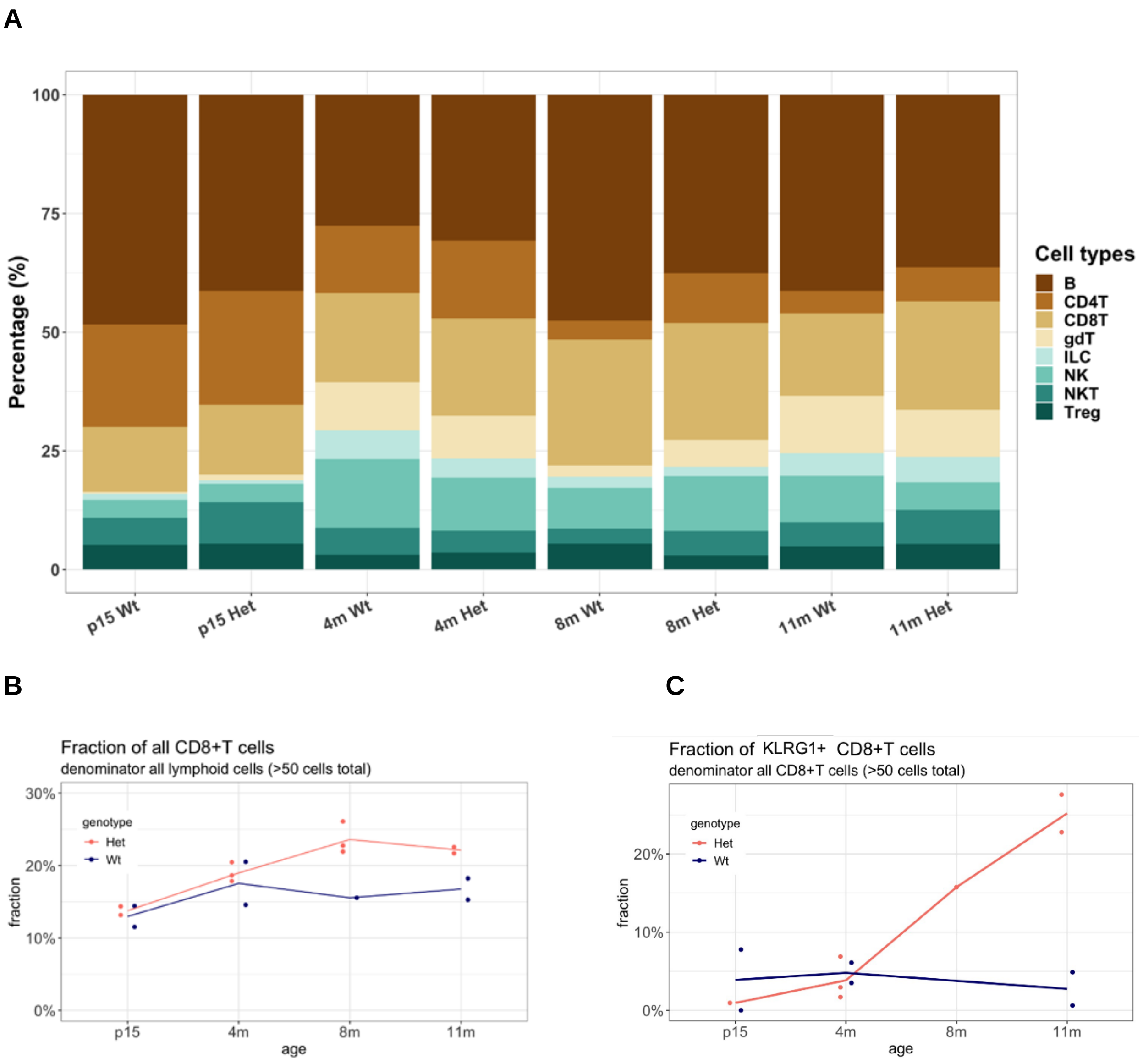

Figure S4. Relative proportion of lymphoid cells in murine lungs as determined by single cell RNA-sequencing data. (A) Fraction of CD4+T cells in lymphoid cells decrease with age in both genotypes. Fraction of gamma delta T cells, innate lymphoid cells, natural killer T cells and natural killer cells among lymphoid cells do not significantly change with age. Combined proportion of CD8+ cells are shown. (B) Fraction of Cd8+T cells among lymphoid cells increases from p15 to 8 months of age and remain elevated in lungs from *Hhip+/-* mice. (C) percentage of Klrg1+terminal effector CD8+T cells among CD8+T cells increases markedly from 4 months to 11 months of age in lungs from *Hhip+/-* mice. Each dot represents one mouse selected with greater than 50 CD8+T cells.

B: B cells, CD4T: CD4+ T cells, ,CD8T: All CD8+ cells, gdT: gamma delta T cells, ILC: innate lymphoid cells ,NK: natural killer cells, NKT: natural killer T cells, Treg: regulatory T cells, Wt: *Hhip+/+*, Het: *Hhip+/-* mice.

A

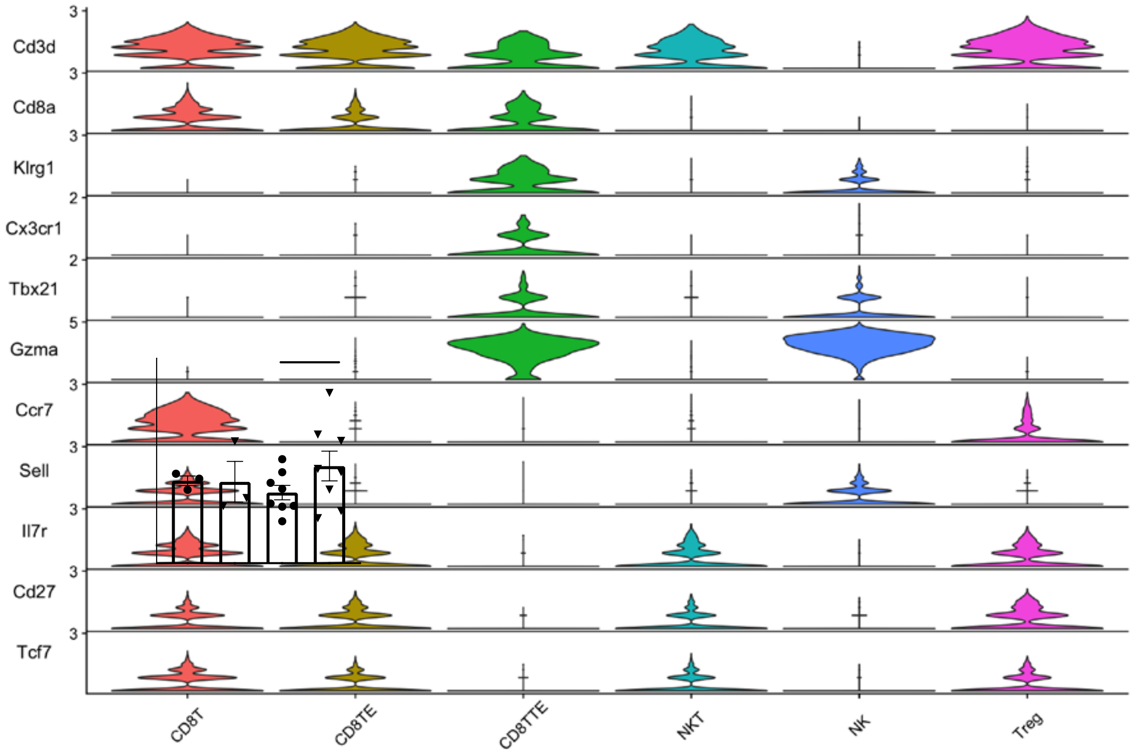
Cd3d
Cd8a
Klrg1
Cx3cr1
Tbx21
Gzma
Ccr7
Sell
Il7r
Cd27
Tcf7
CD8T
CD8TE
CD8TTE
NKT
NK
Treg

B

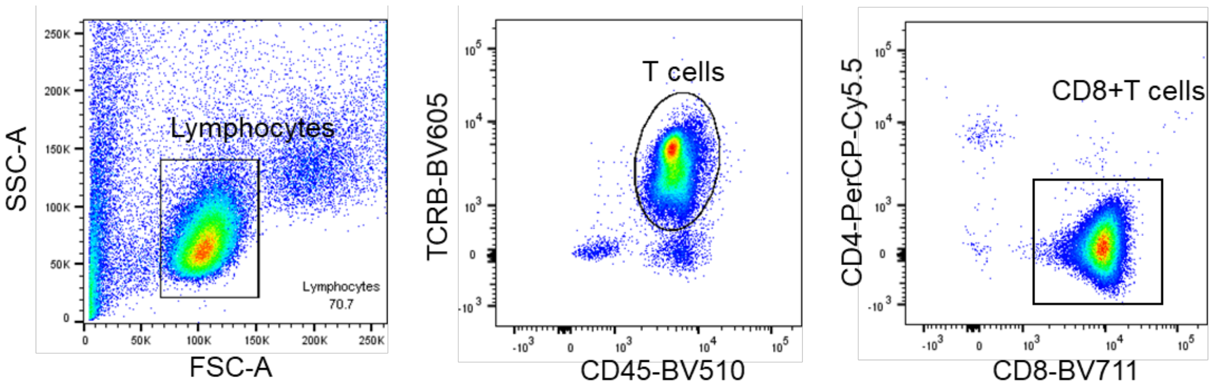
SSC-A
FSC-A
Lymphocytes
Lymphocytes
70.7
TCRB-BV605
CD45-BV510
T cells
CD4-PerCP-Cy5.5
CD8-BV711
CD8+T cells

C

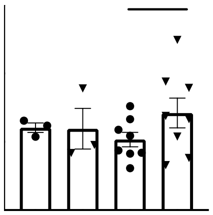

Figure S5. Expression of T cell markers in T cell subsets and FACS gating strategy. (A) Violin plot showing expression of genes in T cell subtypes from *Hhip+/-* and *Hhip+/+* mice combined. Effector markers (Klrg1, Cx3cr1, Tbx21, Gzma) are expressed in CD8 terminal effector cells and NK cells, while memory markers (Ccr7, Sell(CD62L), Il7r, Cd27, Tcf27) are expressed in naïve CD8+T cells, effector memory CD8+ T cells and regulatory T cells. NK cells lack expression of T cell marker Cd3d and Cd8a. (B) Representative FACS gating strategy for CD8+T cells analyzed in the main Figure XX. (C) Geometric mean of KLRG1 fluorescence intensity analyzed by FACS in the main Figure XX from *Hhip+/-* and *Hhip+/+* mice (N=3-8 mice/group). Means+/- SD are shown. Wilcoxon matched pairs signed rank test. CD8T: CD8+T cell (naïve), CD8TE: CD8+effector memory T cell, CD8TTE: CD8+terminal effector cell, NKT: natural killer T cell, NK: natural killer cell, T reg: regulatory T cell.

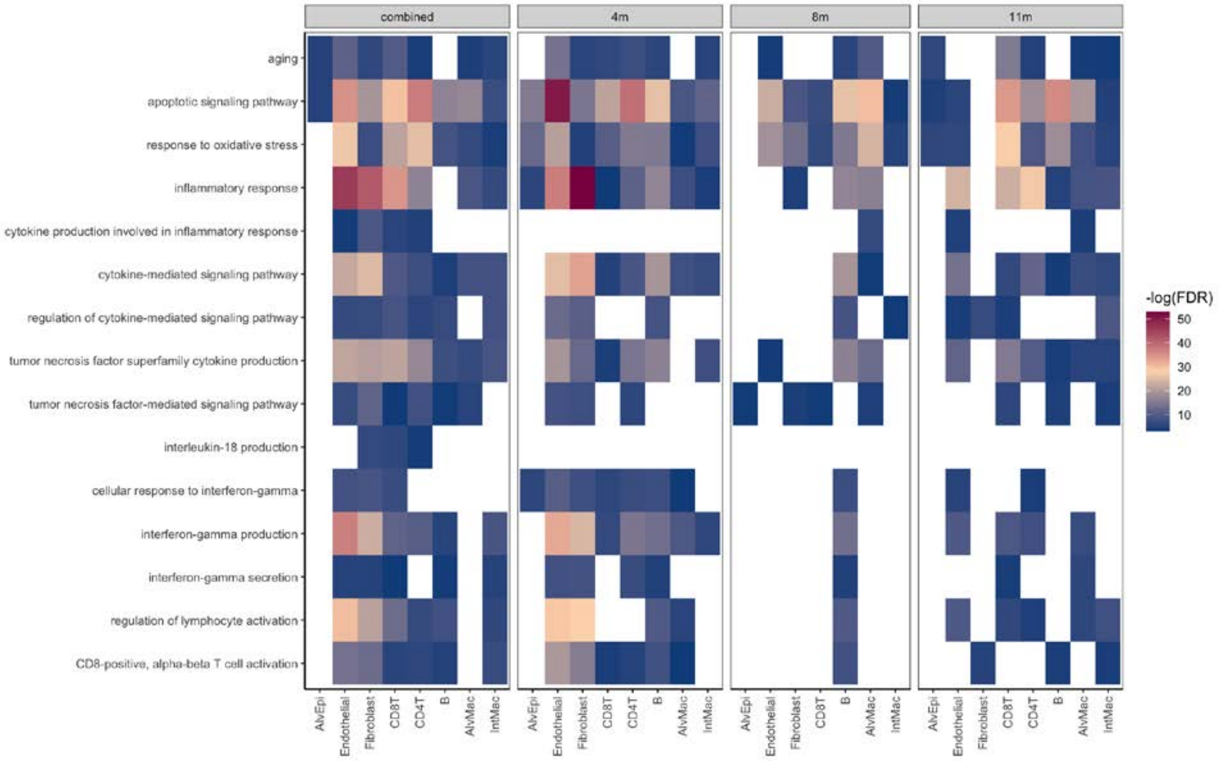

Figure S6. Enrichment of GO: biologic process pathways in upregulated genes in *Hhip* haploinsufficient mice compared to wild type littermate mice identified by g:Profiler(2). Cell type-specific upregulated genes from *Hhip+/-* mice are enriched for common inflammatory pathways including cytokine mediated signaling pathway, interleukin-18-mediated signaling pathway, interferon-gamma, tumor necrosis factor-mediated signaling pathway. FDR: false discovery rate, AlvEpi: alveolar epithelial cells, CD8T: all CD8+ cells, AlvMac: alveolar macrophages, IntMac: interstitial macrophages.

A

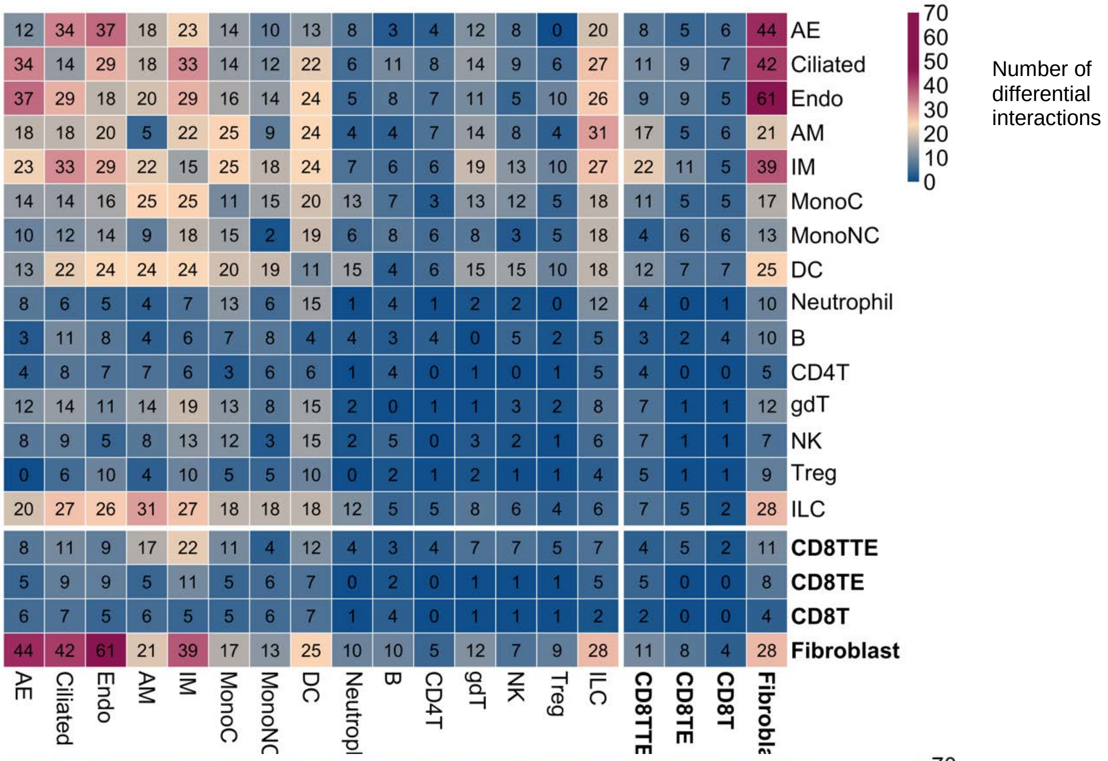

AE
Ciliated
Endo
AM
IM
MonoC
MonoNC
DC
Neutrophil
B
CD4T
gdT
NK
Treg
ILC
CD8TTE
CD8TE
CD8T
Fibroblast
70
60
50
40
30
20
10
0
Number of differential interactions

B

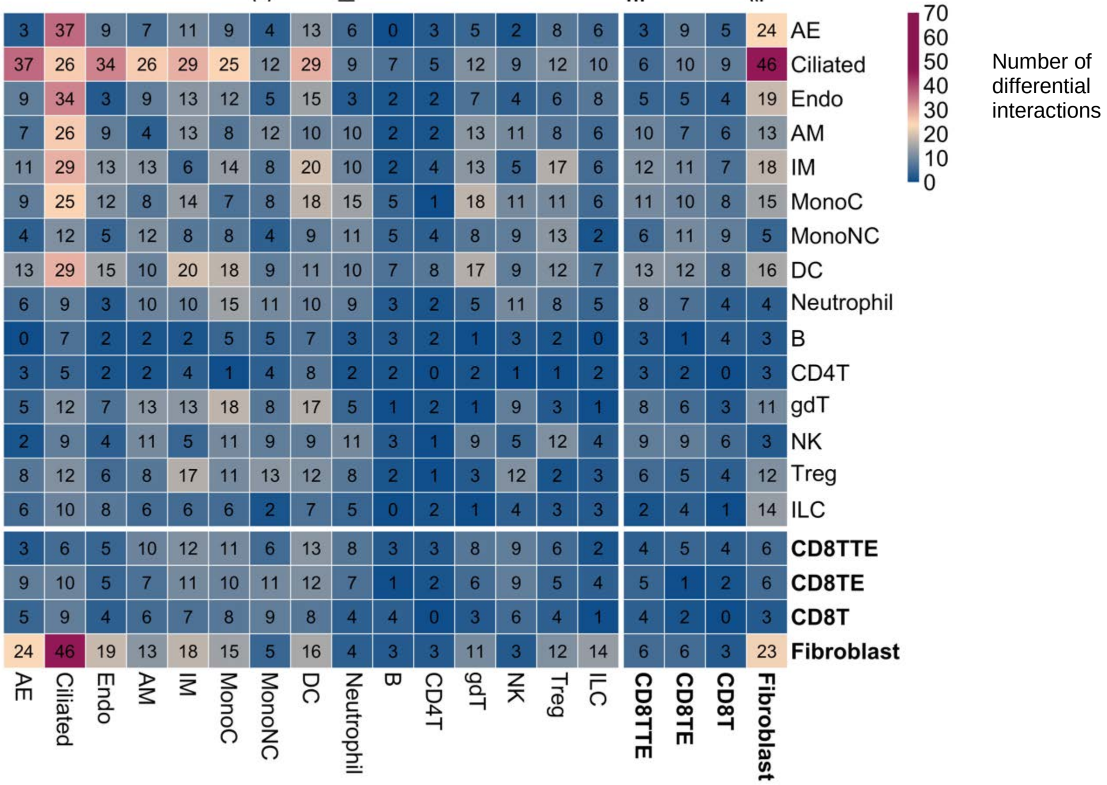

AE
Ciliated
Endo
AM
IM
MonoC
MonoNC
DC
Neutrophil
B
CD4T
gdT
NK
Treg
ILC
CD8TTE
CD8TE
CD8T
Fibroblast
70
60
50
40
30
20
10
0
Number of differential interactions

C

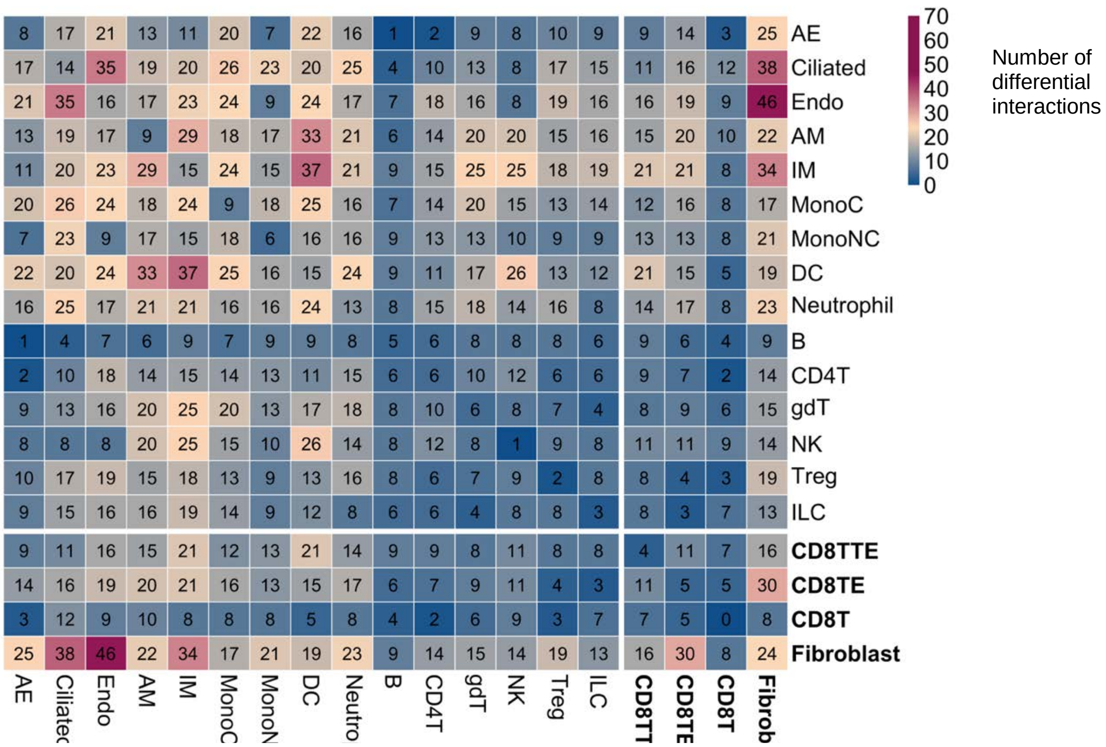
8 17 21 13 11 20 7 22 16 1 2 9 8 10 9 9 14 3 25 AE
17 14 35 19 20 26 23 20 25 4 10 13 8 17 15 11 16 12 38 Ciliated
21 35 16 17 23 24 9 24 17 7 18 16 8 19 16 16 19 9 46 Endo
13 19 17 9 29 18 17 33 21 6 14 20 20 15 16 15 20 10 22 AM
11 20 23 29 15 24 15 37 21 9 15 25 25 18 19 21 21 8 34 IM
20 26 24 18 24 9 18 25 16 7 14 20 15 13 14 12 16 8 17 MonoC
7 23 9 17 15 18 6 16 16 9 13 13 10 9 9 13 13 8 21 MonoNC
22 20 24 33 37 25 16 15 24 9 11 17 26 13 12 21 15 5 19 DC
16 25 17 21 21 16 16 24 13 8 15 18 14 16 8 14 17 8 23 Neutrophil
1 4 7 6 9 7 9 9 8 5 6 8 8 8 6 9 6 4 9 B
2 10 18 14 15 14 13 11 15 6 6 10 12 6 6 9 7 2 14 CD4T
9 13 16 20 25 20 13 17 18 8 10 6 8 7 4 8 9 6 15 gdT
8 8 8 20 25 15 10 26 14 8 12 8 1 9 8 11 11 9 14 NK
10 17 19 15 18 13 9 13 16 8 6 7 9 2 8 8 4 3 19 Treg
9 15 16 16 19 14 9 12 8 6 6 4 8 8 3 8 3 7 13 ILC
9 11 16 15 21 12 13 21 14 9 9 8 11 8 8 4 11 7 16 CD8TTE
14 16 19 20 21 16 13 15 17 6 7 9 11 4 3 11 5 5 30 CD8TE
3 12 9 10 8 8 8 5 8 4 2 6 9 3 7 7 5 0 8 CD8T
25 38 46 22 34 17 21 19 23 9 14 15 14 19 13 16 30 8 24 Fibroblast
AE Ciliatec Endo AM IM MonoC MonoN DC Neutro B CD4T gdT NK Treg ILC CD8TT CD8TE CD8T Fibrob
70 60 50 40 30 20 10 0
Number of differential interactions

D

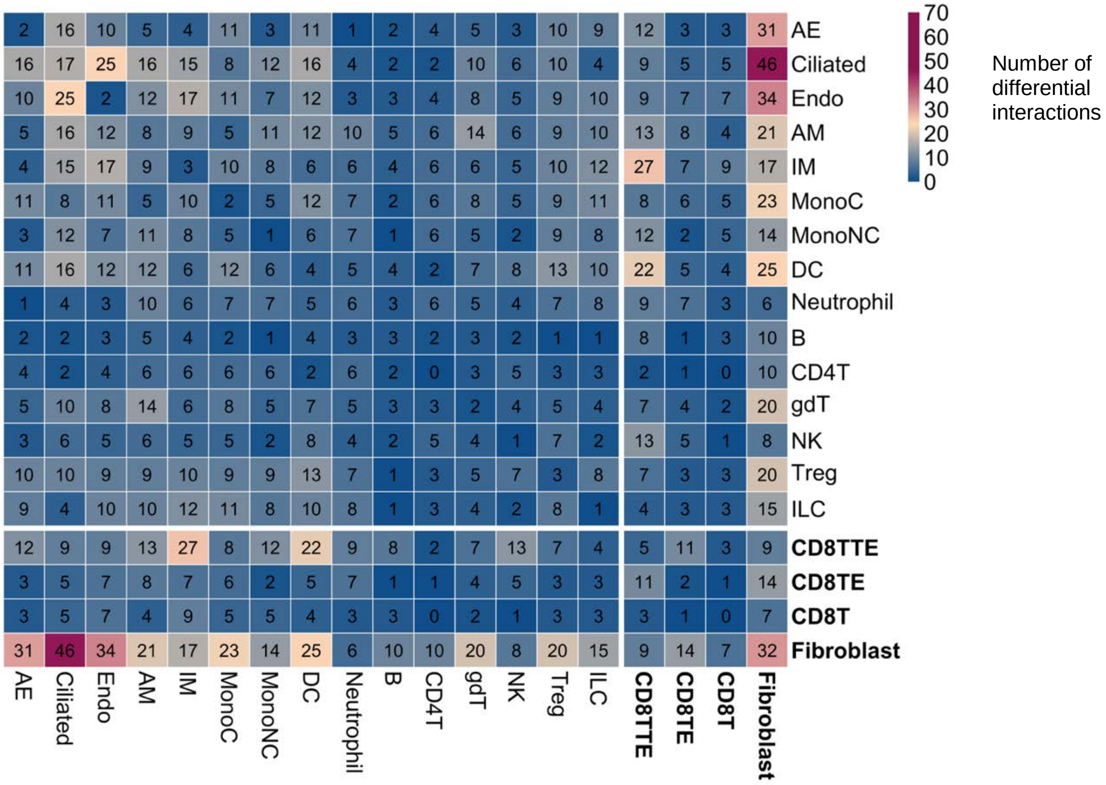
2 16 10 5 4 11 3 11 1 2 4 5 3 10 9 12 3 3 31 AE
16 17 25 16 15 8 12 16 4 2 2 10 6 10 4 9 5 5 46 Ciliated
10 25 2 12 17 11 7 12 3 3 4 8 5 9 10 9 7 7 34 Endo
5 16 12 8 9 5 11 12 10 5 6 14 6 9 10 13 8 4 21 AM
4 15 17 9 3 10 8 6 6 4 6 6 5 10 12 27 7 9 17 IM
11 8 11 5 10 2 5 12 7 2 6 8 5 9 11 8 6 5 23 MonoC
3 12 7 11 8 5 1 6 7 1 6 5 2 9 8 12 2 5 14 MonoNC
11 16 12 12 6 12 6 4 5 4 2 7 8 13 10 22 5 4 25 DC
1 4 3 10 6 7 7 5 6 3 6 5 4 7 8 9 7 3 6 Neutrophil
2 2 3 5 4 2 1 4 3 3 2 3 2 1 1 8 1 3 10 B
4 2 4 6 6 6 6 2 6 2 0 3 5 3 3 2 1 0 10 CD4T
5 10 8 14 6 8 5 7 5 3 3 2 4 5 4 7 4 2 20 gdT
3 6 5 6 5 5 2 8 4 2 5 4 1 7 2 13 5 1 8 NK
10 10 9 9 10 9 9 13 7 1 3 5 7 3 8 7 3 3 20 Treg
9 4 10 10 12 11 8 10 8 1 3 4 2 8 1 4 3 3 15 ILC
12 9 9 13 27 8 12 22 9 8 2 7 13 7 4 5 11 3 9 CD8TTE
3 5 7 8 7 6 2 5 7 1 1 4 5 3 3 11 2 1 14 CD8TE
3 5 7 4 9 5 5 4 3 3 0 2 1 3 3 3 1 0 7 CD8T
31 46 34 21 17 23 14 25 6 10 10 20 8 20 15 9 14 7 32 Fibroblast
AE Ciliated Endo AM IM MonoC MonoNC DC Neutrophil B CD4T gdT NK Treg ILC CD8TTE CD8TE CD8T Fibroblast
70 60 50 40 30 20 10 0
Number of differential interactions

Figure S7. Differential receptor-ligand pair interactions analysis by genotype across different cell types in a given age. The number of alveolar type 1 cells, capillary endothelial and intermediate monocytes in p15 samples and the number of lymphatic endothelial cells, club cells, and basophils across all age points were too small to be included for receptor-ligand interaction anlaysis. (A) p15 samples, (B) 4-month samples, (C) 8- month samples, (D) 11-month samples. The number inside each block indicates number of different receptor-ligand pairs in *Hhip+/-* compared to *Hhip+/+*; Scale bar indicates number of receptor-ligand pairs. AE: alveolar epithelial cell, Endo: endothelial cell, AM: alveolar macrophage, IM: interstitial macrophage, MonoC: classical monocyte, MonoNC: non-classcial monocyte, DC: dendritic cell, B: B cell, gdT: gamma delta T cell, NK: natural killer cell, Treg: regulatory T cell, ILC: innate lymphoid cell, CD8TE: CD8+ effector memory T cell, CD8TTE: CD8+terminal effector T cell.

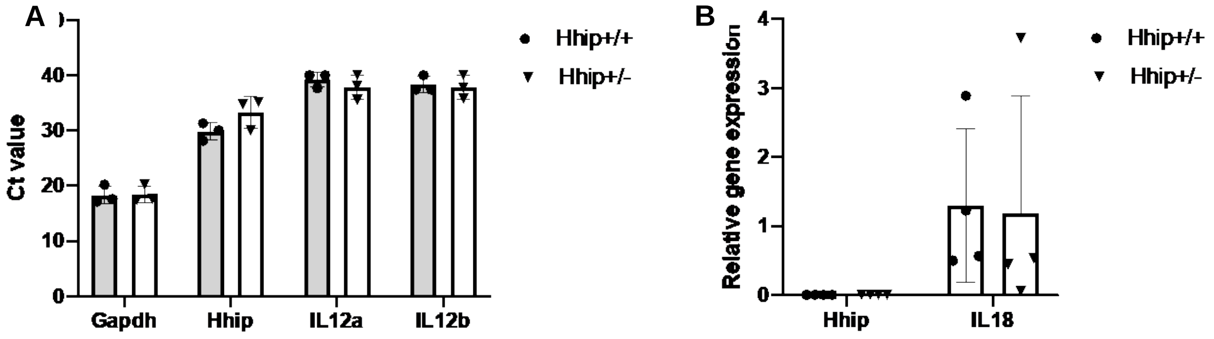

Figure S8. Expression of specific genes by cell type. (A) Cycle threshold (Ct) values of a housekeeping gene (*Gapdh*), *Hhip*, *Il12a* and *Il12b* from real-time PCR measured in lung fibroblasts. Lung fibroblasts were isolated from *Hhip+/+* and *Hhip+/-* mice (n=3 each). (B) *Il18* expression in alveolar macrophages measured by qPCR from *Hhip+/+* and *Hhip+/-* mice. Expression of *Hhip* is not detectable in alveolar macrophages, compared to reference gene *TBP*. IL-18 expression is not different between *Hhip+/+* and *Hhip+/-* mice (n= 4 each). A-B: Data are represented as means ± SD. Each point represents individual biological replicate.

Online supplementary files

Supplementary File 1 lists marker genes specific to individual cell clusters and fibroblast subclusters.
Supplementary File 2 lists differentially expressed genes by genotype combining all age points (combined), and by individual age points.

Supplementary Reference

1. Aran D et al. Reference-based analysis of lung single-cell sequencing reveals a transitional profibrotic macrophage. *Nat. Immunol.* 2019;20(2):163–172.
2. Raudvere U et al. g:Profiler: a web server for functional enrichment analysis and conversions of gene lists (2019 update). *Nucleic Acids Res.* 2019;47(W1):W191–W198.
